# *Diacylglycerol lipase* regulates lifespan and oxidative stress response by inversely modulating TOR signaling in *Drosophila* and *C. elegans*

**DOI:** 10.1111/acel.12232

**Published:** 2014-05-30

**Authors:** Yen-Hung Lin, Yi-Chun Chen, Tzu-Yu Kao, Yi-Chun Lin, Tzu-En Hsu, Yi-Chun Wu, William W Ja, Theodore J Brummel, Pankaj Kapahi, Chiou-Hwa Yuh, Lin-Kwei Yu, Zhi-Han Lin, Ru-Jing You, Yi-Ting Jhong, Horng-Dar Wang

**Affiliations:** 1 Institute of Biotechnology, National Tsing Hua UniversityHsinChu, 30013, Taiwan; 2 Institute of Molecular and Cellular Biology, National Taiwan UniversityTaipei, 10617, Taiwan; 3 Department of Metabolism and Aging, The Scripps Research InstituteJupiter, FL, 33458, USA; 4 Department of Biology, Long Island UniversityBrookville, NY, 11548, USA; 5 Buck Institute for Research on AgingNovato, CA, 94945, USA; 6 Institute of Molecular and Genomic Medicine, National Health Research InstitutesZhunan, Miaoli County, 35053, Taiwan; 7 Department of Life Science, National Tsing Hua UniversityHsinChu, 30013, Taiwan; 8 Institute of Systems Neuroscience, National Tsing Hua UniversityHsinChu, 30013, Taiwan

**Keywords:** aging, diacylglycerol, *diacylglycerol kinase*, metabolism, phosphatidic acid, S6 kinase

## Abstract

Target of rapamycin (TOR) signaling is a nutrient-sensing pathway controlling metabolism and lifespan. Although TOR signaling can be activated by a metabolite of diacylglycerol (DAG), phosphatidic acid (PA), the precise genetic mechanism through which DAG metabolism influences lifespan remains unknown. DAG is metabolized to either PA via the action of DAG kinase or 2-arachidonoyl-*sn*-glycerol by diacylglycerol lipase (DAGL). Here, we report that in *Drosophila* and *Caenorhabditis elegans,* overexpression of *diacylglycerol lipase* (*DAGL*/*inaE*/*dagl-1*) or knockdown of *diacylglycerol kinase* (*DGK*/*rdgA*/*dgk-5*) extends lifespan and enhances response to oxidative stress. Phosphorylated S6 kinase (p-S6K) levels are reduced following these manipulations, implying the involvement of TOR signaling. Conversely, *DAGL*/*inaE*/*dagl-1* mutants exhibit shortened lifespan, reduced tolerance to oxidative stress, and elevated levels of p-S6K. Additional results from genetic interaction studies are consistent with the hypothesis that DAG metabolism interacts with TOR and S6K signaling to affect longevity and oxidative stress resistance. These findings highlight conserved metabolic and genetic pathways that regulate aging.

## Introduction

Longevity is regulated by conserved signaling pathways that modulate aging-associated stress responses (Haigis & Yankner, 2010; Lapierre & Hansen, 2012). The insulin/IGF-1 (IIS) and target of rapamycin (TOR) signaling pathways have been implicated in aging in diverse organisms including yeast, flies, worms, and mammals (Kapahi *et al*., 2010; Kenyon, 2010). TOR is a widely conserved serine/threonine kinase which acts as a nutrient sensor to regulate cell growth, translational control, ribosome biogenesis, autophagy, and metabolism (Wullschleger *et al*., 2006; Stanfel *et al*., 2009; Alic & Partridge, 2011; Zoncu *et al*., 2011). Reduction of TOR activity extends lifespan in many species (Vellai *et al*., 2003; Jia *et al*., 2004; Kapahi *et al*., 2004; Kaeberlein *et al*., 2005; Hansen *et al*., 2007; Harrison *et al*., 2009; Selman *et al*., 2009). Reduction in the activity of S6 protein kinase, a downstream signaling component in the TOR pathway, also leads to lifespan extension and resistance to age-related pathologies in mice (Selman *et al*., 2009), while the activation of Rheb-TOR signaling activity reduces oxidative stress tolerance and hastens emergence of age-related phenotypes in *Drosophila* (Patel & Tamanoi, 2006).

Diacylglycerol (DAG) is an important lipid metabolic intermediate involved in complex signaling pathways (Carrasco & Merida, 2007). DAG can be hydrolyzed by DAG lipase (DAGL) to become 2-arachidonoyl-*sn*-glycerol (2-AG) or modified by DAG kinase (DGK) resulting in its conversion to phophatidic acid (PA) for phosphoinositide turnover (Cai *et al*., 2009; Raghu & Hardie, 2009). PA, as well as the attenuation DAG levels in the cell membrane, affects numerous intracellular signaling pathways, including those regulating cell growth, differentiation, and membrane trafficking (Merida *et al*., 2008). PA can bind to mammalian TOR (mTOR) and promote mTORC1 and mTORC2 formation, which in turn induce the TOR signaling pathway (Toschi *et al*., 2009; Foster, 2013) and lead to elevated phosphorylation levels of S6K and 4EBP (Fang *et al*., 2001).

Previously, we have shown that a multi-stress screening strategy can be used to identify genes or mutants involved in the regulation of longevity (Wang *et al*., 2004, 2012; Liu *et al*., 2009). Here, we report the characterization of one such gene, identified in a *Drosophila* multi-stress resistant strain *DAGL*/*inaE*^*EP1101*^. This EP-element generated line is long-lived and resistant to oxidative stress. *DAGL*/*inaE*^*EP1101*^ shows upregulation of *DAGL*/*inaE,* a homolog of *diacylglycerol lipase*, and reduced levels of phosphorylated S6 kinase (p-S6K), consistent with the hypothesis that *DAGL*/*inaE* up-regulation causes a reduction in TOR signaling. Conversely, a second mutant with reduced *DAGL*/*inaE* expression, *DAGL*/*inaE*^*KG08585*^, displays shortened lifespan, reduced tolerance to oxidative stress and elevated levels of p-S6K. Genetic manipulation of *DAGL*/*inaE*, *rdgA*, or *S6K*^*KQ*^ (a dominant-negative form of S6 kinase) also suggest that reduced TOR signaling mediates the effects of *DAGL*/*inaE* overexpression on lifespan and stress resistance. Using *Caenorhabditis elegans*, we show that, as in flies, the nematode ortholog of *DAGL*/*inaE*, *F42G9.6* (herein named *dagl-1*), also regulates lifespan and oxidative stress response via TOR. We propose that *DAGL*/*inaE* and *DGK* regulate competing branches of pathways that metabolize DAG, ultimately resulting in altered PA levels, which in turn modulate TOR signaling. Collectively, our results show the modulation of longevity and oxidative stress response through conserved pathways that alter TOR signaling in *Drosophila* and *C. elegans*.

## Results

### *Diacylglycerol lipase* regulates longevity and oxidative stress response in *Drosophila*

**Figure 1:**
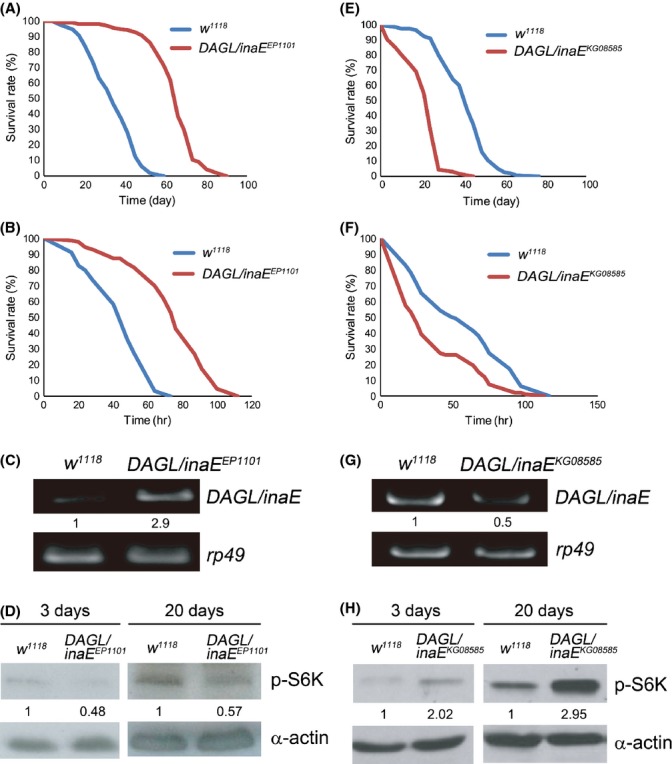
*DAGL*/*inaE* expression regulates lifespan and oxidative stress response, and negatively correlates with phosphorylated S6 kinase (p-S6K) levels in *Drosophila* (A–D). The *Drosophila DAGL*/*inaE*^*EP1101*^ mutant, which exhibits up-regulation of *DAGL*/*inaE*, shows extended lifespan, enhanced oxidative stress response and reduced levels of p-S6K. (A) Lifespan of *DAGL*/*inaE*^*EP1101*^ (mean = 64 d, *n* = 306, red line) and *w*^*1118*^ (mean = 37 d, *n* = 273, blue line). (B) Survival under 10 mm paraquat-induced oxidative stress of *DAGL*/*inaE*^*EP1101*^ (mean = 74 h, *n* = 87, red line) and *w*^*1118*^ (mean = 44 h, *n* = 69, blue line). (C) RT-PCR analysis shows a nearly 200% increase in *DAGL*/*inaE* levels in *DAGL*/*inaE*^*EP1101*^ compared to that of *w*^*1118*^. (D) Levels of p-S6K are decreased in *DAGL*/*inaE*^*EP1101*^ compared to that of *w*^*1118*^ in 3-d and 20-d old flies. (E–H) The *DAGL*/*inaE*^*KG08585*^ mutant, which exhibits down-regulation of *DAGL*/*inaE*, shows shortened lifespan, reduced oxidative stress resistance and increased levels of p-S6K. (E) Lifespan of *DAGL*/*inaE*^*KG08585*^ (mean = 21 d, *n* = 254, red line) and *w*^*1118*^ (mean = 41 d, *n* = 300, blue line). (F) Survival under 10 mm paraquat-induced oxidative stress of *DAGL*/*inaE*^*KG08585*^ (mean = 37 h, *n* = 100, red line) and *w*^*1118*^ (mean = 55 h, *n* = 100, blue line). (G) RT-PCR analysis shows a 50% decrease in *DAGL*/*inaE* levels in *DAGL*/*inaE*^*KG08585*^ compared to that of *w*^*1118*^. RT-PCR results are normalized to *rp49* as an internal control (C, G). (H) Levels of p-S6K are increased in *DAGL*/*inaE*^*KG08585*^ compared to that of *w*^*1118*^ in 3-d and 20-d old flies. α-actin was used as an internal control (D, H).

In a screen for long-lived mutants with enhanced resistance to simultaneous oxidative stress and starvation, we identified an EP-element insertion mutant *DAGL*/*inaE*^*EP1101*^ with a 66% increase (*P* < 0.001) in mean survival time compared to that of the control fly *w*^*1118*^ (Fig. S1, Supporting information). The outcrossed *DAGL*/*inaE*^*EP1101*^ line was 72% longer lived than the control (Fig. 1A) and was similarly more resistant to oxidative stress induced by paraquat (Fig. 1B). To identify the target gene in *DAGL*/*inaE*^*EP1101*^ responsible for lifespan extension and stress resistance, we performed a plasmid rescue and verified that a single *EP*-element insertion was present in the 5′ un-translated region of *DAGL*/*inaE*. The *EP*-element insertion in *DAGL*/*inaE*^*EP1101*^ disrupts the binding site of a transcriptional repressor Tailless (Gui *et al*., 2011). Semi-quantitative RT-PCR analysis revealed a threefold increase of *DAGL*/*inaE* mRNA levels in *DAGL*/*inaE*^*EP1101*^ compared with the control (Fig. 1C). *DAGL*/*inaE* encodes diacylglycerol lipase (DAGL), which metabolizes DAG to 2-AG (Leung *et al*., 2008). Since increased *DAGL*/*inaE* expression extends lifespan and enhances resistance to oxidative stress (Fig. 1A–C), we asked whether *DAGL*/*inaE*^*KG08585*^, a mutant with reduced *DAGL*/*inaE* expression (Fig. 1G), would have the opposite phenotypes. As expected, *DAGL*/*inaE*^*KG08585*^ exhibited a 50% decrease (*P* < 0.001) in mean lifespan and a 34% reduction (*P* < 0.001) in mean survival time on oxidative stress compared to *w*^*1118*^ (Fig. 1E,F). Together, the results suggest that *DAGL*/*inaE* regulates lifespan and oxidative stress resistance in *Drosophila*.

### Overexpression of *DAGL*/*inaE* and knockdown of *rdgA* similarly extend lifespan

To determine whether overexpression of *DAGL*/*inaE* is sufficient to extend lifespan and increase oxidative stress resistance, we generated transgenic flies expressing either the 2214-nt long isoform *DAGL/inaE-PD* cDNA (*UAS-DAGL*/*inaE-PD*) or the 1935-nt short isoform *DAGL/inaE-PA* cDNA (*UAS-DAGL*/*inaE-PA*). Since *DAGL/inaE* expresses mainly in adult fly brain, eye, and thoracic-abdominal ganglion according to the data from FlyAtlas (Chintapalli *et al*., 2007), thus we used *GMR-Gal4* (eye and thoracic-abdominal ganglion Gal4 driver), *Appl-Gal4* (neuronal Gal4 driver), *hs-Gal4,* and *da-Gal4* (ubiquitous Gal4 drivers) to express either *UAS-DAGL*/*inaE-PD* or *UAS-DAGL*/*inaE-PA* to determine if overexpression of *DAGL*/*inaE* would also enhance lifespan and oxidative stress response. In all cases, expression of either *UAS-DAGL*/*inaE-PD* or *UAS-DAGL*/*inaE-PA* by those drivers extended mean lifespan (Table S1, Supporting information) and enhanced oxidative stress resistance (Table S2, Supporting information). These results suggest that neurons are a target tissue for lifespan extension and oxidative stress resistance by *DAGL*/*inaE* overexpression. Since overexpression of both isoforms resulted in similar outcomes, we used only *UAS-DAGL*/*inaE-PD* in all subsequent experiments and hereafter refer to it as *UAS-DAGL*/*inaE*.

Diacylglycerol can be converted to 2-AG by DAGL or metabolized to form phosphatidic acid (PA) by DAG kinase (encoded by *retinal degeneration A* (*rdgA*) in *Drosophila* (Hardie, 2003). In mammalian systems PA is reported to activate target of rapamycin (mTOR) kinase resulting in elevated levels of 4EBP and phosphorylated S6K (Fang *et al*., 2001). Thus, we hypothesize that the enhanced longevity of *DAGL*/*inaE*^*EP1101*^ resulted from reduced TOR signaling, since *DAGL*/*inaE* overexpression shunts more DAG into 2-AG and it should also lower PA levels (Fig. S2, Supporting information). To examine this possibility, we measured the levels of phosphorylated S6 kinase (p-S6K), a downstream molecular marker of TOR signaling. Levels of p-S6K were reduced by 50% and 40% in young and old *DAGL*/*inaE*^*EP1101*^ flies, respectively, relative to levels in *w*^*1118*^ (Fig. 1D). Conversely, in the short-lived *DAGL*/*inaE*^*KG08585*^ p-S6K levels were elevated by 1.5- and threefold in young and old flies, respectively (Fig. 1H).

**Figure 2:**
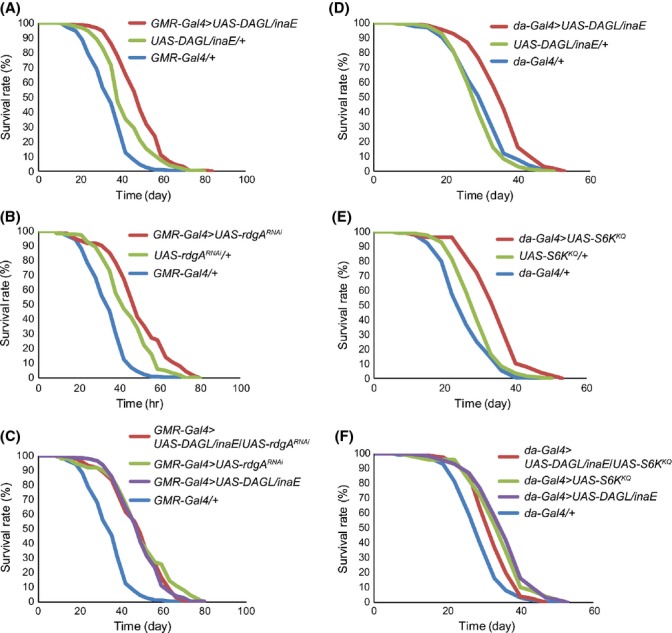
Genetic interaction between *DAGL/inaE*, *rdgA*, and *S6K* on lifespan. (A) Overexpression of *DAGL*/*inaE* driven by *GMR-Gal4* (*GMR-Gal4* > *UAS-DAGL*/*inaE*, mean = 49 d, *n* = 206) extends lifespan compared to controls harboring either genetic element alone (*GMR-Gal4/+*, mean = 34 d, *n* = 258; *UAS-DAGL*/*inaE/+*, mean = 42 d, *n* = 292). (B) Knockdown of *rdgA* by RNAi (*GMR-Gal4>UAS-rdgA*^*RNA*^^*i*^, mean = 50 d, *n* = 62) also extends lifespan compared to controls (*GMR-Gal4/+*, mean = 34 d, *n* = 258; *UAS-rdgA*^*RNA*^^*i*^*/+*, mean = 44, *n* = 173). (C) Lifespan extension by simultaneous overexpression of *DAGL*/*inaE* and knockdown of *rdgA* (*GMR-Gal4>UAS-DAGL*/*inaE* /*UAS-rdgA*^*RNA*^^*i*^, mean = 48 d, *n* = 203) is similar to either manipulation alone (*GMR-Gal4>UAS-DAGL*/*inaE*, mean = 49 d, *n* = 206; *GMR-Gal4>UAS-rdgA*^*RNA*^^*i*^, mean = 50 d, *n* = 62). (D) Overexpression of *DAGL*/*inaE* driven by *da-Gal4* (*da-Gal4* > *UAS-DAGL*/*inaE*, mean = 36 d, *n* = 210) extends lifespan compared to controls (*da-Gal4/+*, mean = 29 d, *n* = 209; *UAS-DAGL*/*inaE/+*, mean = 30 d, *n* = 232) at 29 °C. (E) Overexpression of *S6K*^*KQ*^, a dominant-negative form of S6K, (*da-Gal4* > *UAS-S6K*^*KQ*^, mean = 35 d, *n* = 50) also extends lifespan compared to controls (*da-Gal4/+*, mean = 29 d, *n* = 209; *UAS-S6K*^*KQ*^*/+*, mean = 26 d, *n* = 129) at 29 °C. (F) Lifespan extension by simultaneous overexpression of both *DAGL*/*inaE* and *S6K*^*KQ*^ (*da-Gal4* > *UAS-DAGL*/*inaE/UAS-S6K*^*KQ*^, mean = 33 d, *n* = 76) does not further extend lifespan compared to either manipulation alone (*da-Gal4* > *UAS-DAGL*/*inaE*, mean = 36 d, *n* = 210) or *S6K*^*KQ*^ (*da-Gal4* > *UAS-S6K*^*KQ*^, mean = 35 d, *n* = 50) at 29 °C.

If longevity resulting from *DAGL*/*inaE* overexpression is due to reduced PA formation and TOR signaling, then the knockdown of *rdgA* (DAG kinase) should produce similar phenotypes. Overexpression of *DAGL*/*inaE* resulted in a 41% increase (*P* < 0.001) in mean lifespan compared to *Gal4* alone and 16% (*P* < 0.001) compared to *UAS* alone (Fig. 2A). Knockdown of *rdgA* also significantly increases mean lifespan by 44% (*P* < 0.001) compared to *Gal4* alone or 12% (*P* < 0.01) compared to *UAS* alone (Fig. 2B). Simultaneous *DAGL*/*inaE* overexpression and *rdgA* knockdown did not further extend lifespan of that achieved by either manipulation independently (Fig. 2C). Similar to *DAGL*/*inaE* overexpression, *rdgA* mutants *rdgA*^*BL33306*^ and *rdgA*^*BL20320*^ also displayed an increase of 53% (*P* < 0.001) and 48% (*P* < 0.001) in mean lifespan and 43% reduction for both in p-S6K levels compared to those in control *w*^*1118*^ (Fig. S3A,B, Supporting information). Together, these results are consistent with the idea that *DAGL*/*inaE* and *rdgA* modulate lifespan via a common pathway.

To examine whether overexpression of *DAGL*/*inaE* extends lifespan via reduced TOR signaling, we overexpressed *DAGL*/*inaE* (*UAS-DAGL*/*inaE*) and the dominant-negative form of *S6K* (*UAS-S6K*^*KQ*^) individually and simultaneously. Overexpression of *DAGL*/*inaE* (*UAS-DAGL*/*inaE*) increases mean lifespan by 22% (*P* < 0.001) compared to *Gal4* alone (Fig. 2D). Overexpression of the dominant-negative form of *S6K* (*UAS-S6K*^*KQ*^) extends mean lifespan by 18% (*P* < 0.001) compared to *Gal4* alone (Fig. 2E). Overexpression of both the dominant-negative form of *S6K* and *DAGL*/*inaE* (*UAS-S6K*^*KQ*^; *UAS-DAGL*/*inaE*) simultaneously extends mean lifespan by 17% (*P* < 0.001) compared to *Gal4* alone, which is similar to the longevity observed by overexpression of either transgene individually. Hence, the effects of the individual manipulations on lifespan are non-additive (Fig. 2F); similar results were also observed in the oxidative stress assay (Fig. S4), supporting the notion that *DAGL*/*inaE* -mediated lifespan extension and oxidative stress resistance are the result of lowered TOR signaling.

### Expression of *DAGL*/*inaE* ortholog *dagl-1* also regulates lifespan and is required for oxidative stress response in *C. elegans*

**Figure 3:**
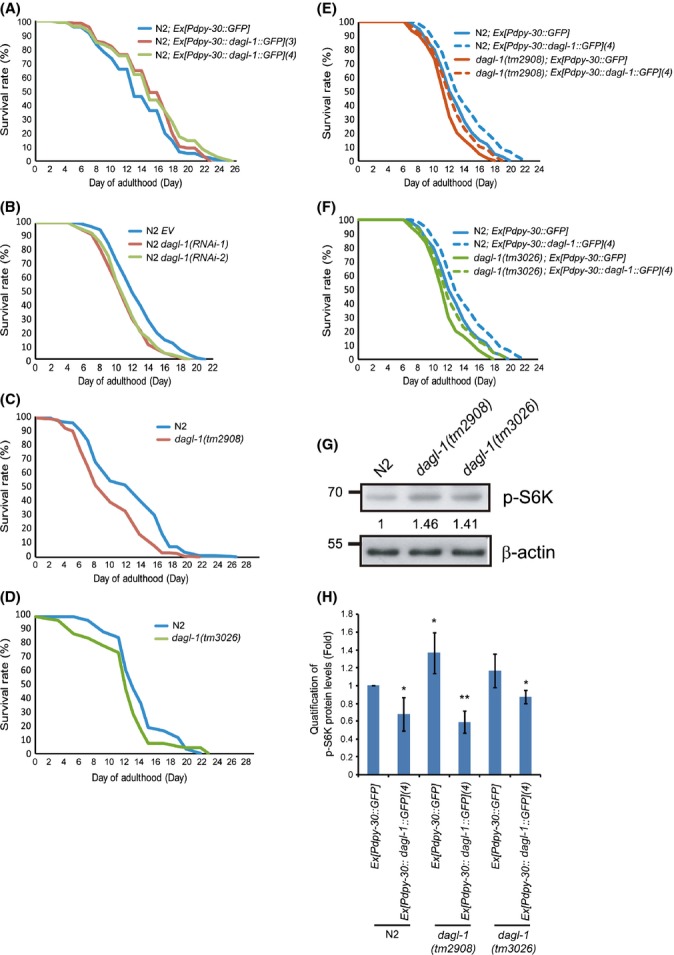
*dagl-1* expression regulates lifespan and negatively correlates with levels of p-S6K in *C. elegans*. (A) Two independent transgenic lines that overexpress *dagl-1* (N2*; Ex[Pdpy::dagl-1::GFP](3)*, red line, and N2*; Ex[Pdpy::dagl-1::GFP](4)*, green line) show extended lifespan compared to the control (N2*; Ex[Pdpy::GFP]*, blue line). (B) N2 worms treated with RNAi against either 5′ (*dagl-1(RNAi-1)*) or 3′ (*dagl-1(RNAi-2)*) coding sequence of *dagl-1* display shortened lifespan compared to the empty vector (EV) control. (C, D) The two *dagl-1* deletion mutants, *dagl-1(tm2908)* and *dagl-1(tm3026)*, exhibit reduced lifespan compared to the control N2. (E, F) Shortened lifespan of *dagl-1(tm2908)* and *dagl-1(tm3026)* can be rescued by transgenic overexpression of *dagl-1*. See also Table S3. (G) Western blot shows elevated levels of p-S6K in *dagl-1(tm2908)* and *dagl-1(tm3026)*. β-actin was used as an internal control. (H) Elevated levels of p-S6K in *dagl-1(tm2908)* and *dagl-1(tm3026)* are reduced by transgenic overexpression of *dagl-1*. Western blots are shown in Fig. S8A.

To determine whether the role of *DAGL*/*inaE* in extending longevity is conserved across species, we performed similar experiments using *C. elegans*. Overexpression of the *DAGL*/*inaE* ortholog *dagl-1* from *C. elegans* driven by a ubiquitous promoter *dpy-30* was achieved using two independent overexpression transgenic lines, N2*; Ex[Pdpy-30::dagl-1::GFP](3)* and N2*; Ex[Pdpy-30::dagl-1::GFP](4)* and comparing the results obtained to the control line N2*; Ex[Pdpy-30::GFP]*. Overexpression of *dagl-1* extends mean lifespan by 12% (*P* < 0.01) and 13% (*P* < 0.001) (Fig. 3A, and Table S3, Supporting information).

Conversely to test whether reduced *dagl-1* expression decreased lifespan, we constructed two RNAi clones (*dagl-1(RNAi-1)* and *dagl-1(RNAi-2)*) targeting the 5′ and 3′ fragment of *dagl-1* coding sequence, respectively. The levels of *dagl-1* expression were reduced by approximately 45% and 24% by feeding N2 worms *E. coli* HT115 harboring *dagl-1(RNAi-1)* and *dagl-1(RNAi-2)*, respectively (Fig. S5, Supporting information). The N2 nematodes treated with *dagl-1(RNAi-1)* and *dagl-1(RNAi-2)* were 14% and 12% shorter lived, relative to the control (Fig. 3B, and Table S3, Supporting information). These results were in agreement with a second approach in which *dagl-1* mutants, *dagl-1(tm2908)* and *dagl-1(tm3026)*, were used. Mean lifespan was reduced by 20% and 13% (Fig. 3C,D, and Table S3, Supporting information). Thus, lower level of *dagl-1* expression is associated with reduced longevity.

To determine whether the effects on longevity in the mutant strains resulted from the reduced expression of *dagl-1*, we generated transgenic lines which overexpressed *dagl-1* (*Pdpy-30::dagl-1)*. The *Pdpy-30::dagl-1,* but not *Pdpy-30::GFP,* transgene significantly rescued the lifespan of the *dagl-1(tm2908)* mutant to the level similar to that seen in the control *N2 [Pdpy-30::GFP]* worms (Fig. 3E, and Table S3, Supporting information). Similar rescue results using the *Pdpy-30::dagl-1* transgene were also observed in the *dagl-1(tm3026)* mutants (Fig. 3F, and Table S3, Supporting information). Together, the data indicate that *dagl-1* expression regulates lifespan in *C. elegans*.

As expected the effects of modulating *dagl-1* expression were similar for both longevity and stress resistance assays. Paraquat-induced oxidative stress was applied to either mutant or RNAi-knockdown *dagl-1* strains. In both cases, the lines with reduced activity were less resistant, *dagl-1(tm2908* or *tm3026)* worms 21–26% less (Fig. S6A, and Table S4, Supporting information) and worms treated with either *dagl-1(RNAi-1)* or *dagl-1(RNAi-2)* 22-27% less resistant (Fig. S6B, and Table S4, Supporting information). Together, the results suggest that *dagl-1* expression is required to respond to oxidative stress in *C. elegans*.

### *dagl-1* modulates lifespan and oxidative stress response through reduced TOR signaling in *C. elegans*

To confirm that *dagl-1* also modulates TOR signaling in *C. elegans*, we first inspected the levels of p-S6K in the *dagl-1* mutant worms compared to N2. In the mutants, p-S6K levels were 41–46% higher than in control worms (Fig. 3G), a result similar to those seen in *Drosophila* (Fig. 1H). The transgenic worms overexpressing *dagl-1* showed significantly lower levels of p-S6K relative to that of the control (Fig. 3H). Moreover, the elevated levels of p-S6K in both *dagl-1* mutant worms were reduced after introducing transgenic *dagl-1* (Fig. 3H). Thus as in *Drosophila,* a clear correlation exists between the effects on TOR signaling and the resulting longevity and stress resistance phenotypes.

### Knockdown of *dgk-5* rescues the shortened lifespan and reduced oxidative stress tolerance in *dagl-1* mutants

**Figure 4:**
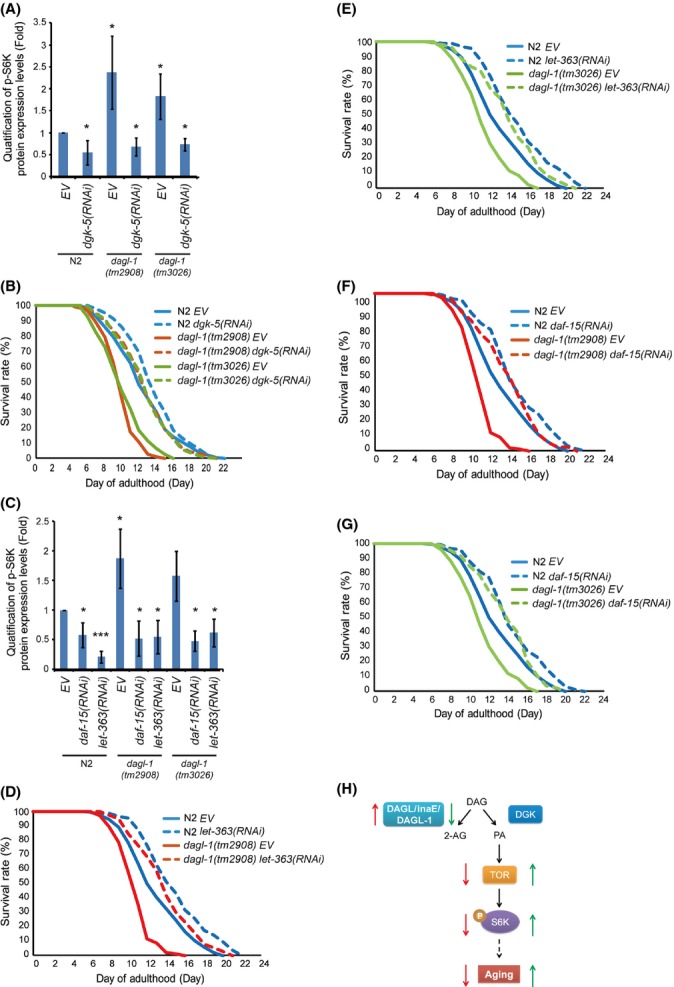
Knockdown of *dgk-5*, *daf-15*, or *let-363* rescues the shortened lifespan and elevated p-S6K levels in *dagl-1* mutants. (A) Elevated levels of p-S6K in *dagl-1(tm2908)* and *dagl-1(tm3026)* are reduced by RNAi knockdown of *dgk-5*. (B) Shortened lifespan of *dagl-1(tm2908)* and *dagl-1(tm3026)* is rescued by RNAi knockdown of *dgk-5*. (C) RNAi knockdown of *daf-15* or *let-363* also reverts the elevated levels of p-S6K observed in *dagl-1(tm2908)* and *dagl-1(tm3026)*. (D–G) Shortened lifespan of *dagl-1* mutants is also rescued by RNAi knockdown of *daf-15* or *let-363*. See also Table S5. All western blots are shown in Fig. S8B and C. (H) Model for *DAGL*/*inaE*/*dagl-1* in regulation of lifespan in *Drosophila* and *C. elegans*. *DAGL*/*inaE*/*dagl-1* overexpression reduces TOR signaling and p-S6K levels to slow aging (red arrows). Hypomorphs of *DAGL*/*inaE*/*dagl-1* increase TOR signaling and p-S6K levels to accelerate aging (green arrows).

Since *dagl-1* mutation may enhance TOR signaling by converting more DAG to PA by diacylglycerol kinase (DGK) (model in Fig. S2), we predicted that the enhancement of TOR signaling in *dagl-1* mutants could be blocked by using RNAi knockdown of the other branch of the pathway, *DGK*/*dgk-5*. In both *dagl-1* mutants p-S6K levels were significantly reduced by RNAi knockdown of *DGK*/*dgk-5* (Fig. 4A). In addition, *DGK*/*dgk-5* RNAi knockdown fully rescued the shortened lifespan (Fig. 4B, and Table S5, Supporting information) and improved the response to oxidative stress in both the *dagl-1* mutant worms (Fig. S6C, D, and Table S4, Supporting information). These results showed that knockdown of *DGK*/*dgk-5* can rescue the shortened lifespan and the reduced oxidative stress tolerance in both *dagl-1* mutants.

As *rdgA* (*Drosophila DGK*) mutants demonstrated increased lifespan and lowered p-S6K levels (Fig. S3A,B, Supporting information), we examined whether *C. elegans dgk-5* mutants also present the similar phenotypes. Intriguingly, *C. elegans dgk-5* mutants *dgk-5(ok2366)* and *dgk-5(gk631)* had an 8% (*P* < 0.05) and 11% (*P* < 0.01) increase in mean lifespan and 31% and 50% decline in p-S6K levels compared to those in control N2, respectively (Fig. S3D,E, Table S6, Supporting information). These results are consistent with the *rdgA* mutant analysis in *Drosophila* and, together they bolster our hypothesis (Fig. S2, Supporting information).

### RNAi knockdown of *Tor/let-363* or *raptor/daf-15* reduces the elevated p-S6K levels, rescues the shortened lifespan and improves the oxidative stress response in *dagl-1* mutants

To further verify that TOR signaling plays a role in *dagl-1*-mediated lifespan and oxidative stress response in *C. elegans*, we examined whether RNAi knockdown of the Tor kinase, *Tor*/*let-363*, blocks the increase in p-S6K levels in the *dagl-1* mutants. As expected, p-S6K levels were dramatically reduced in *dagl-1* mutant worms treated with *Tor/let-363* RNAi-containing bacteria (Fig. 4C). Raptor binds to TOR to form TOR complex 1 and regulates TOR downstream signaling (Wullschleger *et al*., 2006). Therefore, we checked whether RNAi knockdown of *raptor/daf-15* expression could also diminish the elevated levels of p-S6K in the *dagl-1* mutants and found that the enhanced p-S6K levels were also significantly reduced in both *dagl-1(tm2908)* and *dagl-1(tm3026)* mutants treated with *raptor/daf-15* RNAi (Fig. 4C). Moreover, treatment of either *Tor/let-363* or *raptor/daf-15* RNAi to *dagl-1(tm2908)* and *dagl-1(tm3026)* mutant worms also rescued their shortened lifespan (Fig. 4D–G, and Table S5, Supporting information).

To determine whether comparable results would be obtained in oxidative stress response, we conducted similar experiments in *dagl-1(tm2908)* and *dagl-1(tm3026)* worms treated with or without *Tor/let-363* or *raptor/daf-15* RNAi under paraquat-induced oxidative stress. In these experiments the reduced survival rates in both *dagl-1(tm2908)* and *dagl-1(tm3026)* mutants were almost completely rescued by of the treatment of either *Tor/let-363* or *raptor/daf-15* RNAi (Fig. S6E,F, and Table S4, Supporting information). Together, the results reveal that the RNAi knockdown of *Tor/let-363* or *raptor/daf-15* not only lowers the elevated p-S6K levels but also rescues the shortened lifespan and partially improves the oxidative stress response in the *dagl-1* mutant worms.

To exclude the possibility that 2-AG itself may also reduce TOR signaling, we exogenously supplemented 2-AG into NIH3T3 and Hep3B cell lines and examined the levels of p-S6K. 2-AG did not cause any reduction in the levels of p-S6K in both NIH3T3 and Hep3B cell lines, while rapamycin treatment dramatically reduced the levels of p-S6K (Fig. S7, Supporting information).

In summary, our parallel analysis using *Drosophila* and *C. elegans* demonstrate that *DAGL/inaE/dagl-1* regulates lifespan and modulates oxidative stress response through inversely modulating TOR signaling (Fig. 4H).

## Discussion

Genetic studies in model organisms have led to the discovery of many genes that can modulate aging. In addition many of these studies suggest that the pathways that control aging have been evolutionarily conserved. TOR signaling is one of the conserved nutrient sensor pathways involved in metabolism, growth, and nutrient sensing, and plays an important role in the regulation of aging from yeast to mammals including humans (Kapahi *et al*., 2010). TOR is proposed to be a lipid sensor that modulates cell growth and proliferation (Foster, 2013). However, we did not observe any changes in the eye and wing sizes (Fig. S9, Supporting information) neither the body size (data not shown) upon *DAGL*/*inaE* overexpression, suggesting that *DAGL*/*inaE* overexpression does not affect developmental growth. Accumulated evidence has shown that lipid metabolism is linked to lifespan regulation (Oldham, 2011; Ackerman & Gems, 2012). In this study, we demonstrated that *diacylglycerol lipase* (*DAGL/inaE/dagl-1*) regulates lifespan and oxidative stress response through TOR signaling in both *Drosophila* and *C. elegans*. Overexpression of *DAGL/inaE/dagl-1* may shunt more DAG toward the production of 2-AG, thereby leaving less DAG available to produce PA, and consequently resulting in reduced TOR signaling. Both in flies and worms, *DAGL/inaE/dagl-1*-mediated lifespan is negatively correlated with levels of p-S6K. Both the shortened lifespan and the elevated levels of p-S6K can be rescued and reverted by the RNAi knockdown of *dgk-5*, *daf-15*, or *let-363* in the *dagl-1* mutants, suggesting that TOR signaling plays a role in *DAGL/inaE/dagl-1* mediated lifespan. In addition, we also showed that both RNAi knockdown of *DGK/rdgA/dgk-5* and their mutants extend lifespan and exhibit reduced level of pS6K in *Drosophila* and *C. elegans*. This is the first demonstration showing that reduced *rdgA* and *dgk-5* expression extend lifespan in *Drosophila* and *C. elegans*. Together, it suggests that genetically altered DAG metabolism may influence PA levels to affect TOR signaling mediated lifespan and stress response.

Lipid homeostasis is critical to aging. Several genes involved in lipid metabolism control lifespan (Ackerman & Gems, 2012; McCormick *et al*., 2012). DAG is a lipid metabolic intermediate as a second messenger involved in complex signaling (Carrasco & Merida, 2007). DAG can activate protein kinase D (PKD). It has been suggested that DGK functions upstream of PKD in the regulation of oxidative-induced intestinal cell injury (Song *et al*., 2008). Thus, genetic manipulation of DGK and PKD should produce similar phenotypes. Indeed, it was reported that *PKD*/*DFK-2* deficiency increases adult lifespan by 40% in *C. elegans* (Feng *et al*., 2007), implying that a lower level of DAG may extend lifespan in *C. elegans*. This is in agreement with our idea that lower DAG levels results in less PA formation, reduced TOR signaling, and thus to an extension of lifespan – an effect mimicked by knockdown of *DGK*/*rdgA*/*dgk-5* both in *Drosophila* and *C. elegans*. It was reported that *Drosophila* microRNA *mir-14* inhibits reaper-dependent cell death and is required for lipid metabolism (Xu *et al*., 2003). Depletion of *mir-14* results in reduced lifespan and lowered stress tolerance and is accompanied with increased levels of triacylglycerol and diacylglycerol and the above phenotypes are reverted upon increasing *mir-14* copy number in *Drosophila*. This suggests that lifespan negatively correlates with DAG level. DAG activation of protein kinase C (PKC) is linked to hepatic insulin resistance, a risk for type 2 diabetes (Jornayvaz & Shulman, 2012). In addition, PKC activity is associated with prefrontal cortical decline in aging and pharmacological inhibition of PKC rescues working memory malfunction in aged rat and increased working memory in aged rhesus monkeys (Brennan *et al*., 2009), indicating accumulated DAG is deleterious to lifespan and health. DAG is a second messenger triggering activation of PKC to enhance calcium influx for the activation of mTORC1. Overexpression of *DAGL*/*inaE* in neurons may result in less DAG levels for lowered PKC activity leading to reduced calcium influx and hence diminished mTORC1 activity to account for the extended lifespan and oxidative stress resistance. Thus, altered lipid metabolism achieved by lowering DAG levels is beneficial to lifespan and stress response.

Phophatidic acid is implicated in the activation of mammalian target of rapamycin (mTOR) and the control of cell growth and differentiation (Fang *et al*., 2001; Merida *et al*., 2008). Overexpression of *DAGL*/*inaE*/*dagl-1* may result in lower level of PA for reduced TOR signaling in extending lifespan. It was reported that the expression of a specific isoform DGKζ, which modulates PA levels, regulates the levels of serum-induced phosphorylation of S6K for mTOR signaling in HEK293 cells (Avila-Flores *et al*., 2005). Interestingly, the closest homologs of DGKζ in *Drosophila* and *C. elegans* are *rdgA* and *dgk-5,* respectively. Our data showed that not only the mutants of *rdgA* and *dgk-5* but also both knockdown of *rdgA* in fly and knockdown of *dgk-5* in worm extend lifespan and reduce the levels of p-S6K. This provides the first *in vivo* evidence that reducing *DGK* extends lifespan via its effect on TOR signaling in both *Drosophila* and *C. elegans*. As we hypothesized that *DAGL* overexpression may result in more 2-AG formation, and 2-AG can be further metabolized to become arachidonic acid, also known as omega-6 polyunsaturated fatty acids, and glycerol. Omega-6 polyunsaturated fatty acids recently have been reported to extend *C. elegans* lifespan via activation of autophagy (O’Rourke *et al*., 2013). Therefore, it is also possible that *DAGL/inaE/dagl-1* overexpression may result in more 2-AG for increased levels of omega-6 polyunsaturated fatty acids to activate autophagy for lifespan extension.

Insulin signaling is a well-studied and conserved pathway that also regulates lifespan (Kenyon, 2010). The interplay between insulin and TOR signaling pathways is well characterized (Hay, 2011). Interestingly, we found that the longevity and oxidative stress resistance of *daf-2* can be partially inhibited by knockdown of *dagl-1*, and increased *dagl-1* expression was detected in a *daf-2* mutant. Two putative daf-16 binding sites were identified in the regulatory region of *dagl-1* (Liu *et al*., 2012). In addition, we also found increased levels of phosphorylated Akt (p-Akt) in the two *dagl-1* mutants compared to N2 (Lin and Wang, unpublished data), suggesting that *dagl-1* plays a role in the lifespan and oxidative stress response of the *daf-2* mutant and insulin signaling may also modulate *dagl-1* expression in *C. elegans*. However, we did not detect any changes in the levels of p-Akt in *DAGL*/*inaE*^*EP1101*^ and *DAGL*/*inaE*^*KG08585*^ compared to *w*^*1118*^ (data not shown). It suggests that there is a discrepancy between *Drosophila* and *C. elegans* in insulin signaling for the *DAGL*/*inaE*/*dagl-1*-mediated lifespan regulation.

In summary, our study shows that *DAGL*/*inaE*/*dagl-1* regulates lifespan and oxidative stress response via negatively modulating TOR signaling in both *Drosophila* and *C. elegans*. Since TOR signaling is a conserved pathway among different species regulating nutrient sensing, cell growth, and aging, our discovery may be relevant in mammals. Our results provide new insights on how the altered genetic regulation of DAG metabolism affects lifespan and stress response and may help in developing therapies to DAG imbalance-related diseases.

## Experimental procedures

### *Drosophila* and *C. elegans* strains and RNAi-expressing bacteria clones

The fly line *DAGL*/*inaE*^*EP1101*^ (Rorth, 1996) was initially identified in a double stress screen in Dr. Seymour Benzer’s laboratory (Caltech, Pasadena, CA, USA). *DAGL*/*inaE*^*KG08585*^, *rdgA*^*BL33306*^*,* and *rdgA*^*BL20320*^ were later obtained from the Bloomington *Drosophila* stock center. All were outcrossed with *w*^*1118*^ for at least six or ten generations to eliminate background effects and the resultant homozygous lines were used for lifespan and oxidative stress assays. *UAS-rdgA*^*RNAi*^ (VDRC #3024) was obtained from the Vienna *Drosophila* RNAi Center (VDRC). All flies were raised on standard Caltech fly food at 25 °C with 65%-humidity and a 12-hour light/dark cycle (Liu *et al*., 2009). The *dagl-1* frame-shift mutant strains, *dagl-1(tm2908)* and *dagl-1(tm3026)*, were obtained from the National Bioresource Project. The *dgk-5(ok2366)* and *dgk-5(gk631)* strains were provided by Dr. Chang-Shi Chen from Taiwan *C. elegans* Core. All nematodes were grown at 20 °C on Nematode Growth Medium (NGM) plates seeded with OP50 for regular culture or with HT115 for RNAi treatment. The RNAi clones targeting *daf-15* and *let-363* were kindly provided by Dr. Ao-Lin Hsu at University of Michigan. Two RNAi plasmids, *dagl-1(RNAi-1)* and *dagl-1(RNAi-2)*, were constructed using the L4440 vector that express double-stranded RNA targeting either the 5′ or 3′ end of *dagl-1* cDNA upon IPTG induction. The 517-nt amplicon of *dagl-1* for *dagl-1(RNAi-1)* and the 570-nt amplicon of *dagl-1* for *dagl-1(RNAi-2)* were PCR amplified by the primer sets (RNAi-1 forward: 5′-GGCAAGTCAATGGTAGTGGA-3′ and RNAi-1 reverse: 5′- CGAAACAACGCTCATCACAT-3′; and RNAi-2 forward: 5′- TTCCGCTTGCCTGTTCTACT-3′ and RNAi-2 reverse: 5′-CCTGCAACAACATCACTTGG-3′) and subcloned into L4440 vector.

### Generation of *DAGL/inaE* transgenic flies and *dagl-1* transgenic worms

To generate the *DAGL*/*inaE* transgenic flies, the 2214-nt long isoform (*inaE-PD*, FlyBase) and the 1935-nt short isoform (*inaE-PA*, FlyBase) of *DAGL*/*inaE* cDNAs based on the information of FlyBase were PCR-amplified using *LD44686* and *GH19816* plasmids as templates and subcloned into the *XhoI*/*BglII* sites of *pINDY6* transgenic vector (Wang *et al*., 2004). The resultant transgenic constructs were verified by DNA sequencing to confirm no mutations in the cDNAs derived from PCR, and later micro-injected into *w*^*1118*^ embryos to generate the transgenic flies, *UAS-DAGL*/*inaE-PD* and *UAS-DAGL*/*inaE-PA*, expressing either the long or the short isoforms of *DAGL*/*inaE* upon Gal4 induction. For *dagl-1* transgenic nematodes, *F42G9.6b* isoform full-length cDNA – which is the most homologous to fly *DAGL*/*inaE*-*PD* gene – was subcloned and fused with GFP driven by the *dpy-30* ubiquitous promoter in the *ps235* vector (Hsu *et al*., 1995). The resultant plasmid, *Pdpy-30::dagl-1::GFP*, was verified by DNA sequencing and micro-injected at a concentration of 20 ng/μl into N2 young adult worms to generate the two independent transgenic worms, N2; *Ex[Pdpy-30::dagl-1::GFP](3)* and N2; *Ex[Pdpy-30::dagl-1::GFP](4)*. The control worms, N2; *Ex[Pdpy-30::GFP]*, were obtained by micro-injecting the control plasmid *Pdpy-30::GFP* into N2. The progeny of the injected animals were screened for GFP expression to establish independent lines.

### Lifespan and oxidative stress assays in *Drosophila* and *C. elegans*

For *Drosophila*, the lifespan assay and paraquat-induced oxidative stress assay for the progeny from specific crosses were carried out as described previously (Liao *et al*., 2008; Liu *et al*., 2009; Wang *et al*., 2012). We found female flies in *DAGL*/*inaE*^*EP1101*^ and *DAGL*/*inaE*^*KG08585*^ showed similar results to males in the lifespan and stress assays and thus only results from male flies were used in this paper. Most experiments were carried out at 25 °C unless otherwise stated. For *C. elegans*, lifespan assays were performed at 20 °C as described previously (Liu *et al*., 2009) but without adding 5′ flourodeoxyuridine (FUdR). N2, *dagl-1*(*tm2908)*, *dagl-1(tm3026)*, N2; *Ex[Pdpy-30::GFP]*, N2; *Ex[Pdpy-30::dagl-1::GFP](3)* and N2; *Ex[Pdpy-30::dagl-1::GFP](4)* worms were grown on NGM plates seeded with *E. coli* OP50 bacteria. For RNAi treatment, worms were placed on NGM plates with *E. coli* HT115 containing the control L4440 plasmid or L4440 expressing dsRNA targeting the specific gene. All the worms were initially transferred daily for the first seven days and later every 2 or 3 days. Dead worms not responding to gentle prodding were scored until all were dead. The oxidative stress assay for worms was conducted at 20 °C. Young adult hermaphrodites were immersed in S-media containing either 10 or 40 mm of paraquat (1,1-dimethyl-4,4-bipyridinium dichloride, Sigma-Aldrich, St. Louis, MO, USA). The number of dead worms was scored every hour until all worms were dead. All experiments were repeated at least three times. Gene expression changes were monitored by RT-PCR and real-time PCR. Statistical differences in survival were calculated by the log-rank test. Differences in oxidative stress resistance were determined by Student’s *t*-test.

### Western blot

Fly heads of specific age for each strain were collected and homogenized in lysis buffer containing protease inhibitor (Cat#: 04693159001, Roche, Indianapolis, IN, USA) and phosphatase inhibitor (Cat#: 04906837001, Roche). Synchronized four-day-old adult worms of each strain, with or without RNAi treatment, were collected in 15ml centrifuge tubes, washed three times by M9 buffer, transferred to new microfuge tubes, and homogenized by lysis buffer containing protease inhibitor and phosphatase inhibitor. In the cell lines, NIH3T3 and Hep3B cells were treated with DMSO as a mock, 10 or 20 μm of 2-AG (Cat#: 1298, TOCRIS, Bristol, UK), or 10 nm Rapamycin as a positive control (Cat#: 553210, Millipore, Billerica, MA, USA). After 24-h incubation, the treated cells were collected in 15-mL centrifuge tubes and the cell pellets were lysed in lysis buffer containing protease inhibitor and phosphatase inhibitor as mentioned above. After homogenization, 2% SDS was added to each sample again and then the sample was vortexed and incubated 5 min at 70 °C. The sample was centrifuged at 13 000 rpm for 10 min at room temperature and the supernatant was transferred into new tube to measure protein concentration. Equal amounts of protein for each sample were loaded and separated in a 12% SDS-PAGE gel, and transferred to a nitrocellulose membrane. The membrane was blocked with 5% BSA in TBST for 1 h, and later incubated with anti-pS6K (Cell Signaling, Billerica, MA, USA, #9209, 1:500 dilution in 5% BSA /1XTBST or Abbomax Inc., #601-030, 1:1000 dilution in 5% BSA /1XTBST), anti-pERK (Epitomics, #1481-1, 1:1000 in 5% BSA/1XTBST), anti-α−actin or β-actin or tubulin (α−actin, Santa Cruz, Dallas, Texas, USA, #SC-1616; β-actin, Spring, #E4554, 1:10 000; tubulin, Epitomics, #1871-1, 1:1000 dilution, in 5% BSA/1XTBST), or anti-GAPDH (Epitomics, #S0011, 1:2000 in 5% BSA/1XTBST) at 4 °C overnight. The membrane then was washed three times with TBST, and incubated with the secondary antibody (goat anti-rabbit IgG, 1:10 000 in 5% BSA/1XTBST) for 2 h at 4 °C, again washed three times with TBST, incubated with ECL reagent (Cat#: RPN 2132, Amersham, GE Healthcare, Fairfield, CT, USA) and exposed to the X-ray film (Kodax, Rochester, NY, USA). The protein image was quantified by ImageJ® to calculate the fold of changes by normalizing each measurement to its control.

### Semi-quantitative RT-PCR and quantitative real-time PCR assays

*Drosophila* total RNA extraction and reverse-transcription following by semi-quantitative polymerase chain reaction (RT-PCR) were described in Wang *et al*. (2004). For *C. elegans*, worms with or without RNAi treatment were collected into 1.5-mL microfuge tube, washed three times by M9 buffer, and lysed by using 1 ml TRIzol® reagent (Life Technologies, Grand Island, NY, USA) to extract RNA. Subsequent procedures were similar to those used for *Drosophila*. Each gene was amplified by gene specific primers (sequences available upon request). The genes *rp49* and *actin* were used as internal controls in the PCR reactions for *Drosophila* and *C. elegans*, respectively. The fold changes for gene expression were calculated, normalized to the internal control, by quantification of the image of the DNA in agarose gel by ImageJ® software. Alternatively, the cDNAs were used as templates in quantitative real-time PCR utilizing SYBR Green PCR Master Mix in the Applied Biosystems 7900HT Fast Real-Time PCR System (7900HT Fast System, Life Technologies). Each gene was amplified with the specific real-time PCR primer set, and was normalized to the control (*rp49* for *Drosophila* and *actin* for *C. elegans*). The relative transcriptional levels of the genes were presented as fold of 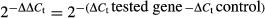. *C*_t_ is the threshold cycle value clarified as the fractional cycle number at the time of target fluorescent signal passed a threshold above baseline.

## Supporting Information

**Fig. S1.** *DAGL*/*inaE*^*EP1101*^ mutant is resistant to the combination of oxidative stress and starvation. **Fig. S2**. Model for *DAGL*/*inaE*/*dagl-1* regulation of lifespan in *Drosophila* and *C. elegans*. **Fig. S3**. *Drosophila rdgA* and *C. elegans dgk-5* mutants exhibit lower p-S6K levels and extended lifespan. **Fig. S4**. Overexpression of both *DAGL*/*inaE* and *S6K*^*KQ*^, a dominant-negative form of S6K, does not further enhance resistance to oxidative stress compared to the overexpression of *DAGL*/*inaE* or *S6K*^*KQ*^ individually. **Fig. S5**. Real-time quantitative PCR analysis shows reduced *dagl-1* expression upon RNAi knockdown in N2 worms. **Fig. S6**. *C. elegans dagl-1* mutants show reduced tolerance to oxidative stress that can be rescued by RNAi knockdown of *dgk-5*, *daf-15*, or *let-363*. **Fig. S7**. Exogenous supplementation of 2-AG does not reduce levels of phosphorylated-S6K (p-S6K) in NIH3T3 and Hep3B cell lines. **Fig. S8**. Western blots for p-S6K. Independent experiments are shown for the results in Figs. **Fig. S9**. No smaller eye or wing size was detected upon *DAGL*/*inaE* overexpression. **Table S1**. Lifespan of *DAGL*/*inaE* transgenic overexpression flies by different Gal4 drivers. **Table S2**. Oxidative stress^g^ response of *DAGL*/*inaE* transgenic overexpression flies by different Gal4 drivers. **Table S3**. Effect of *dagl-1* expression on lifespan of N2 and *dagl-1(tm2908)* and *dagl-1(tm3026)* in *C. elegans*. **Table S4**. Effect of *dagl-1* expression and knockdown of *dgk-5, let-363, daf-15* on oxidative stress response of N2 and *dagl-1(tm2908)* and *dagl-1(tm3026)* in *C. elegans*. **Table S5**. Effect of *dgk-5, let-363,* and *daf-15* RNAi knockdown on the lifespan of N2 and *dagl-1(tm2908)* and *dagl-1(tm3026)* in *C. elegans*. **Table S6**. The lifespan of N2, *dgk-5(ok2366)* and *dgk-5(gk631)* in *C. elegans*. **Table S7.** Effect of *dagl-1* expression on lifespan of N2 and *dagl-1(tm2908)* and *dagl-1(tm3026)* in *C. elegans*. (the three separate data for Table S3). **Table S8**. Effect of *dagl-1* expression and knockdown of *dgk-5, let-363, daf-15* on oxidative stress response of N2 and *dagl-1(tm2908)* and *dagl-1(tm3026)* in *C. elegans*. (the three separate data for Table S4). **Table S9**. Effect of *dgk-5, let-363,* and *daf-15* RNAi knockdown on the lifespan of N2 and *dagl-1(tm2908)* and *dagl-1(tm3026)* in *C. elegans*. (the three separate data for Table S5). **Table S10**. The lifespan of N2, *dgk-5(ok2366)* and *dgk-5(gk631)* in *C. elegans*. (the three separate data for Table S6).

## References

[b1] Ackerman D, Gems D (2012). The mystery of *C. elegans* aging: an emerging role for fat. Distant parallels between *C. elegans* aging and metabolic syndrome?. BioEssays.

[b2] Alic N, Partridge L (2011). Death and dessert: nutrient signalling pathways and ageing. Curr. Opin. Cell Biol.

[b3] Avila-Flores A, Santos T, Rincon E, Merida I (2005). Modulation of the mammalian target of rapamycin pathway by diacylglycerol kinase-produced phosphatidic acid. J. Biol. Chem.

[b4] Brennan AR, Yuan P, Dickstein DL, Rocher AB, Hof PR, Manji H, Arnsten AF (2009). Protein kinase C activity is associated with prefrontal cortical decline in aging. Neurobiol. Aging.

[b5] Cai J, Abramovici H, Gee SH, Topham MK (2009). Diacylglycerol kinases as sources of phosphatidic acid. Biochim. Biophys. Acta.

[b6] Carrasco S, Merida I (2007). Diacylglycerol, when simplicity becomes complex. Trends Biochem. Sci.

[b7] Chintapalli VR, Wang J, Dow JA (2007). Using FlyAtlas to identify better *Drosophila melanogaster* models of human disease. Nat. Genet.

[b8] Fang Y, Vilella-Bach M, Bachmann R, Flanigan A, Chen J (2001). Phosphatidic acid-mediated mitogenic activation of mTOR signaling. Science.

[b9] Feng H, Ren M, Chen L, Rubin CS (2007). Properties, regulation, and in vivo functions of a novel protein kinase D: *Caenorhabditis elegans* DKF-2 links diacylglycerol second messenger to the regulation of stress responses and life span. J. Biol. Chem.

[b10] Foster DA (2013). Phosphatidic acid and lipid-sensing by mTOR. Trends Endocrinol. Metab.

[b11] Gui H, Li ML, Tsai CC (2011). A tale of tailless. Dev. Neurosci.

[b12] Haigis MC, Yankner BA (2010). The aging stress response. Mol. Cell.

[b13] Hansen M, Taubert S, Crawford D, Libina N, Lee SJ, Kenyon C (2007). Lifespan extension by conditions that inhibit translation in *Caenorhabditis elegans*. Aging Cell.

[b14] Hardie RC (2003). TRP channels in *Drosophila* photoreceptors: the lipid connection. Cell Calcium.

[b15] Harrison DE, Strong R, Sharp ZD, Nelson JF, Astle CM, Flurkey K, Nadon NL, Wilkinson JE, Frenkel K, Carter CS, Pahor M, Javors MA, Fernandez E, Miller RA (2009). Rapamycin fed late in life extends lifespan in genetically heterogeneous mice. Nature.

[b16] Hay N (2011). Interplay between FOXO, TOR, and Akt. Biochim. Biophys. Acta.

[b17] Hsu DR, Chuang PT, Meyer BJ (1995). DPY-30, a nuclear protein essential early in embryogenesis for *Caenorhabditis elegans* dosage compensation. Development.

[b18] Jia K, Chen D, Riddle DL (2004). The TOR pathway interacts with the insulin signaling pathway to regulate *C. elegans* larval development, metabolism and life span. Development.

[b19] Jornayvaz FR, Shulman GI (2012). Diacylglycerol activation of protein kinase Cepsilon and hepatic insulin resistance. Cell Metab.

[b20] Kaeberlein M, Powers RW, Steffen KK, Westman EA, Hu D, Dang N, Kerr EO, Kirkland KT, Fields S, Kennedy BK (2005). Regulation of yeast replicative life span by TOR and Sch9 in response to nutrients. Science.

[b21] Kapahi P, Zid BM, Harper T, Koslover D, Sapin V, Benzer S (2004). Regulation of lifespan in *Drosophila* by modulation of genes in the TOR signaling pathway. Curr. Biol.

[b22] Kapahi P, Chen D, Rogers AN, Katewa SD, Li PW, Thomas EL, Kockel L (2010). With TOR, less is more: a key role for the conserved nutrient-sensing TOR pathway in aging. Cell Metab.

[b23] Kenyon CJ (2010). The genetics of ageing. Nature.

[b24] Lapierre LR, Hansen M (2012). Lessons from *C. elegans*: signaling pathways for longevity. Trends Endocrinol. Metab.

[b25] Leung H-T, Tseng-Crank J, Kim E, Mahapatra C, Shino S, Zhou Y, An L, Doerge RW, Pak WL (2008). DAG lipase activity is necessary for TRP channel regulation in *Drosophila* photoreceptors. Neuron.

[b26] Liao PC, Lin HY, Yuh CH, Yu LK, Wang HD (2008). The effect of neuronal expression of heat shock proteins 26 and 27 on lifespan, neurodegeneration, and apoptosis in *Drosophila*. Biochem. Biophys. Res. Commun.

[b27] Liu YL, Lu WC, Brummel TJ, Yuh CH, Lin PT, Kao TY, Li FY, Liao PC, Benzer S, Wang HD (2009). Reduced expression of alpha-1,2-mannosidase I extends lifespan in *Drosophila melanogaster* and *Caenorhabditis elegans*. Aging Cell.

[b28] Liu H, Wang X, Wang HD, Wu J, Ren J, Meng L, Wu Q, Dong H, Kao TY, Ge Q, Wu ZX, Yuh CH, Shan G (2012). *Escherichia coli* noncoding RNAs can affect gene expression and physiology of *Caenorhabditis elegans*. Nat. Commun.

[b29] McCormick M, Chen K, Ramaswamy P, Kenyon C (2012). New genes that extend *Caenorhabditis elegans*’ lifespan in response to reproductive signals. Aging Cell.

[b30] Merida I, Avila-Flores A, Merino E (2008). Diacylglycerol kinases: at the hub of cell signalling. Biochem J.

[b31] Oldham S (2011). Obesity and nutrient sensing TOR pathway in flies and vertebrates: functional conservation of genetic mechanisms. Trends Endocrinol. Metab.

[b32] O’Rourke EJ, Kuballa P, Xavier R, Ruvkun G (2013). omega-6 Polyunsaturated fatty acids extend life span through the activation of autophagy. Genes Dev.

[b33] Patel PH, Tamanoi F (2006). Increased Rheb-TOR signaling enhances sensitivity of the whole organism to oxidative stress. J. Cell Sci.

[b34] Raghu P, Hardie RC (2009). Regulation of *Drosophila* TRPC channels by lipid messengers. Cell Calcium.

[b35] Rorth P (1996). A modular misexpression screen in *Drosophila* detecting tissue-specific phenotypes. Proc. Natl Acad. Sci. USA.

[b36] Selman C, Tullet JM, Wieser D, Irvine E, Lingard SJ, Choudhury AI, Claret M, Al-Qassab H, Carmignac D, Ramadani F, Woods A, Robinson IC, Schuster E, Batterham RL, Kozma SC, Thomas G, Carling D, Okkenhaug K, Thornton JM, Partridge L, Gems D, Withers DJ (2009). Ribosomal protein S6 kinase 1 signaling regulates mammalian life span. Science.

[b37] Song J, Li J, Mourot JM, Evers BM, Chung DH (2008). Diacylglycerol kinase regulation of protein kinase D during oxidative stress-induced intestinal cell injury. Biochem. Biophys. Res. Commun.

[b38] Stanfel MN, Shamieh LS, Kaeberlein M, Kennedy BK (2009). The TOR pathway comes of age. Biochim. Biophys. Acta.

[b39] Toschi A, Lee E, Xu L, Garcia A, Gadir N, Foster DA (2009). Regulation of mTORC1 and mTORC2 complex assembly by phosphatidic acid: competition with rapamycin. Mol. Cell. Biol.

[b40] Vellai T, Takacs-Vellai K, Zhang Y, Kovacs AL, Orosz L, Muller F (2003). Genetics: influence of TOR kinase on lifespan in *C. elegans*. Nature.

[b41] Wang H-D, Kazemi-Esfarjani P, Benzer S (2004). Multiple-stress analysis for isolation of *Drosophila* longevity genes. Proc. Natl Acad. Sci. USA.

[b42] Wang CT, Chen YC, Wang YY, Huang MH, Yen TL, Li H, Liang CJ, Sang TK, Ciou SC, Yuh CH, Wang CY, Brummel TJ, Wang HD (2012). Reduced neuronal expression of ribose-5-phosphate isomerase enhances tolerance to oxidative stress, extends lifespan, and attenuates polyglutamine toxicity in *Drosophila*. Aging Cell.

[b43] Wullschleger S, Loewith R, Hall MN (2006). TOR signaling in growth and metabolism. Cell.

[b44] Xu P, Vernooy SY, Guo M, Hay BA (2003). The *Drosophila* microRNA Mir-14 suppresses cell death and is required for normal fat metabolism. Curr. Biol.

[b45] Zoncu R, Efeyan A, Sabatini DM (2011). mTOR: from growth signal integration to cancer, diabetes and ageing. Nat. Rev. Mol. Cell Biol.

