# Supplementary material for: Diacylglycerol lipase regulates lifespan and oxidative stress response by inversely modulating TOR signaling in Drosophila and C. elegans
Source: Aging Cell. 2014 May 30;13(4):755–64. doi: 10.1111/acel.12232 (PMC4116436; doi:10.1111/acel.12232)
Supplement: Supplementary file 1 — Fig. S1. DAGL/inaEEP1101 mutant is resistant to the combination of oxidative stress and starvation. Fig. S2. Model for DAGL/inaE/dagl-1 regulation of lifespan in Drosophila and C. elegans. Fig. S3. Drosophila rdgA and C. elegans dgk-5 mutants exhibit lower p-S6K levels and extended lifespan. Fig. S4. Overexpression of both DAGL/inaE and S6KKQ, a dominant-negative form of S6K, does not further enhance resistance to oxidative stress compared to the overexpression of DAGL/inaE or S6KKQ individually. Fig. S5. Real-time quantitative PCR analysis shows reduced dagl-1 expression upon RNAi knockdown in N2 worms. Fig. S6. C. elegans dagl-1 mutants show reduced tolerance to oxidative stress that can be rescued by RNAi knockdown of dgk-5, daf-15, or let-363. Fig. S7. Exogenous supplementation of 2-AG does not reduce levels of phosphorylated-S6K (p-S6K) in NIH3T3 and Hep3B cell lines. Fig. S8. Western blots for p-S6K. Independent experiments are shown for the results in Figs. Fig. S9. No smaller eye or wing size was detected upon DAGL/inaE overexpression. Table S1. Lifespan of DAGL/inaE transgenic overexpression flies by different Gal4 drivers. Table S2. Oxidative stressg response of DAGL/inaE transgenic overexpression flies by different Gal4 drivers. Table S3. Effect of dagl-1 expression on lifespan of N2 and dagl-1(tm2908) and dagl-1(tm3026) in C. elegans. Table S4. Effect of dagl-1 expression and knockdown of dgk-5, let-363, daf-15 on oxidative stress response of N2 and dagl-1(tm2908) and dagl-1(tm3026) in C. elegans. Table S5. Effect of dgk-5, let-363, and daf-15 RNAi knockdown on the lifespan of N2 and dagl-1(tm2908) and dagl-1(tm3026) in C. elegans. Table S6. The lifespan of N2, dgk-5(ok2366) and dgk-5(gk631) in C. elegans. Table S7. Effect of dagl-1 expression on lifespan of N2 and dagl-1(tm2908) and dagl-1(tm3026) in C. elegans. (the three separate data for Table S3). Table S8. Effect of dagl-1 expression and knockdown of dgk-5, let-363, daf-15 on oxidative stress respo [file acel0013-0755-sd1.docx]

**Supporting Information:**


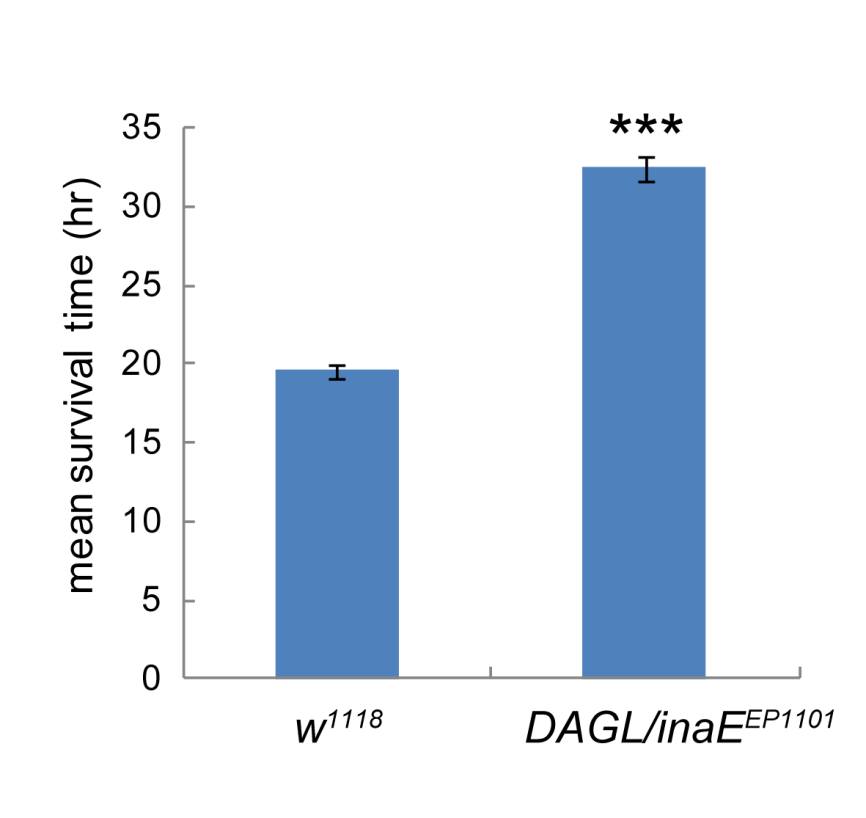


Fig. S1. *DAGL*/*inaE^EP1101^* mutant is resistant to the combination of oxidative stress and starvation. *DAGL*/*inaE^EP1101^* (mean = 32.4 ± 0.8 h, *n* = 90) shows an increase of 66% (*P* < 0.001) in mean survival time compared to the control *w^1118^* (mean = 19.5 ± 0.5 h, *n* = 96) under the dual stress (10 mM paraquat dissolved in water only). (***, *P* < 0.001).


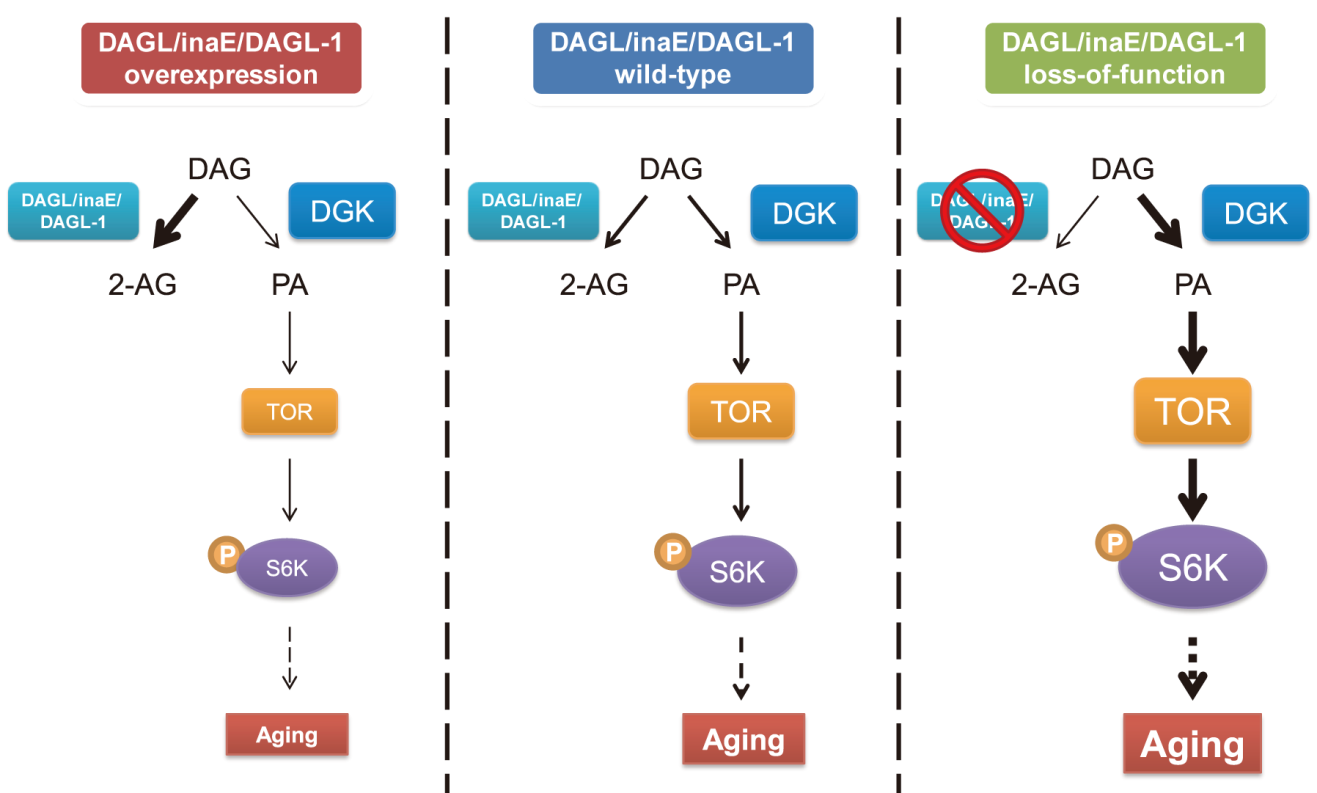


Fig. S2. Model for *DAGL*/*inaE*/*dagl-1* regulation of lifespan in *Drosophila* and *C. elegans*. In the left panel, *DAGL*/*inaE*/*dagl-1* overexpression increases the conversion of DAG to 2-AG, thereby reducing the available DAG for conversion to PA. This ultimately reduces TOR signaling and levels of p-S6K to slow aging, such as in the mutants *DAGL*/*inaE^EP1101^*, *N2; Ex[Pdpy-30::dagl-1::GFP](3)*, and *N2; Ex[Pdpy-30::dagl-1::GFP] (4)*, compared to control animals such as *w^1118^* and N2 in *Drosophila* or *C. elegans*, respectively, which have an unaltered metabolism (middle panel). In the right panel, *DAGL*/*inaE*/*dagl-1* hypomorphs or loss-of-function mutants may divert more DAG to be metabolized to PA, enhancing TOR signaling and elevating p-S6K levels to ultimately accelerate aging, such as in the mutants *DAGL*/*inaE^KG08585^*, *dagl-1(tm2908)*, and *dagl-1(tm3026)* in *Drosophila* and *C. elegans*, respectively. The thickness of the arrows for DAG metabolism and the sizes of TOR, p-S6K, and ‘Aging’ represent their rates and activities, respectively, relative to the wild-type in the middle panel.


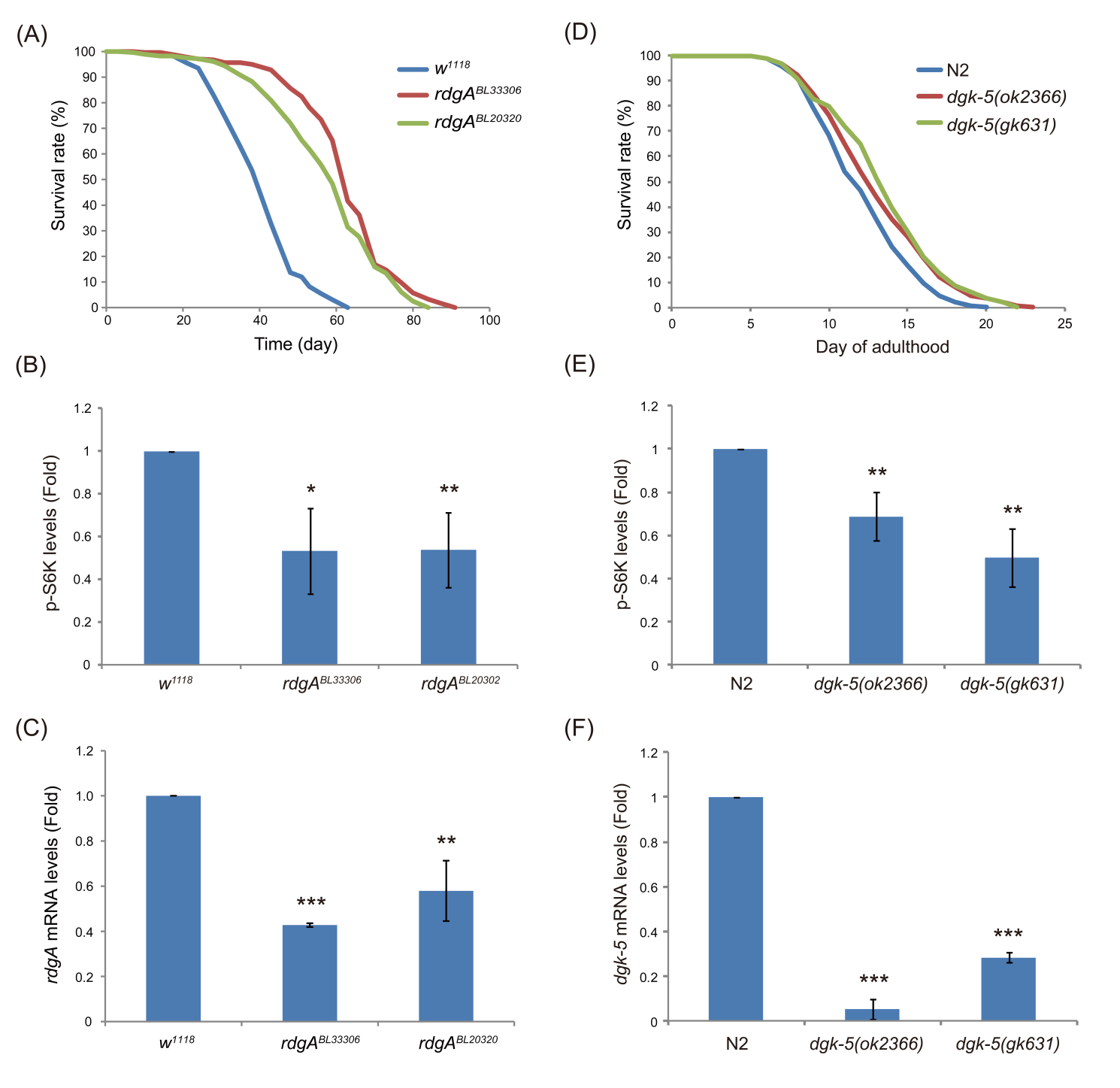


Fig. S3. *Drosophila* *rdgA* and *C. elegans* *dgk-5* mutants exhibit lower p-S6K levels and extended lifespan. *Drosophila* *rdgA* mutants *rdgA^BL33306^* and *rdgA^BL20320^* displayed (A) increased mean lifespan of 55% (*rdgA^BL33306^*, mean = 66.2 ± 3.3 d, *n* = 279, red line, *P* < 0.001) and 48% (*rdgA^BL20320^*, mean = 57.8 ± 5.1 d, *n* = 283, green line, *P* < 0.001) compared to control *w^1118^* (mean = 40.4 ± 1.9 d, *n* = 293, blue line), (B) 43% reductions in p-S6K levels (*P* < 0.05 for *rdgA^BL33306^*; *P* < 0.01 for *rdgA^BL20320^*, compared to *w^1118^*), and (C) decreased *rdgA* mRNA levels by 57% and 42%, respectively, by real-time quantitative PCR compared to that in the control *w^1118^*. Similarly, *C. elegans dgk-5* mutants *dgk-5(ok2366)* and *dgk-5(gk631)* exhibited (D) mean lifespan extension of 8% (mean = 13.3 ± 0.3 d, *n* = 287, red line, *P* < 0.05) and 11% (mean = 13.6 ± 0.4 d, *n* = 207, green line, *P* < 0.01) compared to N2 (mean = 12.3 ± 0.3 d, *n* = 216, blue line) (see also Table S6), (E) reduced p-S6K levels of 31% (*P* < 0.01) and 50% (*P* < 0.01) (western blots shown in Fig. S8D), and (F) reduced *dgk-5* mRNA levels by 94% (*P* < 0.001) and 72% (*P* < 0.001), respectively, compared to that in the control N2.


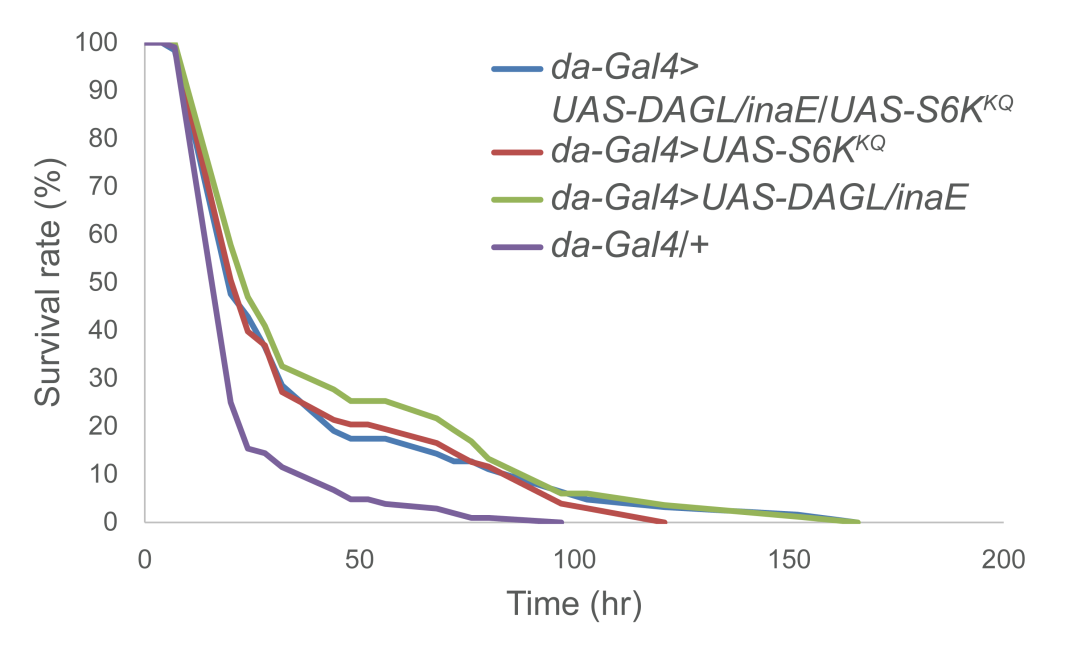


Fig. S4. Overexpression of both *DAGL*/*inaE* and *S6K^KQ^*, a dominant negative form of S6K, does not further enhance resistance to oxidative stress compared to the overexpression of *DAGL*/*inaE* or *S6K^KQ^* individually. Overexpression of both *DAGL*/*inaE* and *S6K^KQ^* simultaneously (*da-Gal4>UAS-DAGL*/*inaE* */UAS-S6K^KQ^*, mean = 38.5 ± 2.4 h, *n* = 63, *P* < 0.001, red line), of *DAGL*/*inaE* (*da-Gal4>UAS-DAGL*/*inaE*, mean = 43.2 ± 2.7 h, *n* = 83, *P* < 0.001, purple line) or of *S6K^KQ^* (*da-Gal4>UAS-S6K^KQ^*, mean = 37.5 ± 4.9 h, *n* = 103, *P* < 0.001, green line) show similarly the same improved survival on paraquat-induced (20 mM) oxidative stress compared to the control (*da-Gal4/*+, mean = 24.9 ± 2.9 h, *n* = 104, blue line). Survival of the three overexpression lines were identical (*da-Gal4>UAS-DAGL*/*inaE* */UAS-S6K^KQ^* versus *da-Gal4*>*UAS-S6K^KQ^*, *P*=0.56; *da-Gal4>UAS-DAGL*/*inaE* */UAS-S6K^KQ^* versus *da-Gal4*>*UAS-DAGL*/*inaE*, *P*=0.139; *da-Gal4*>*UAS-S6K^KQ^* versus *da-Gal4*>*UAS-DAGL*/*inaE* , *P*=0.384).


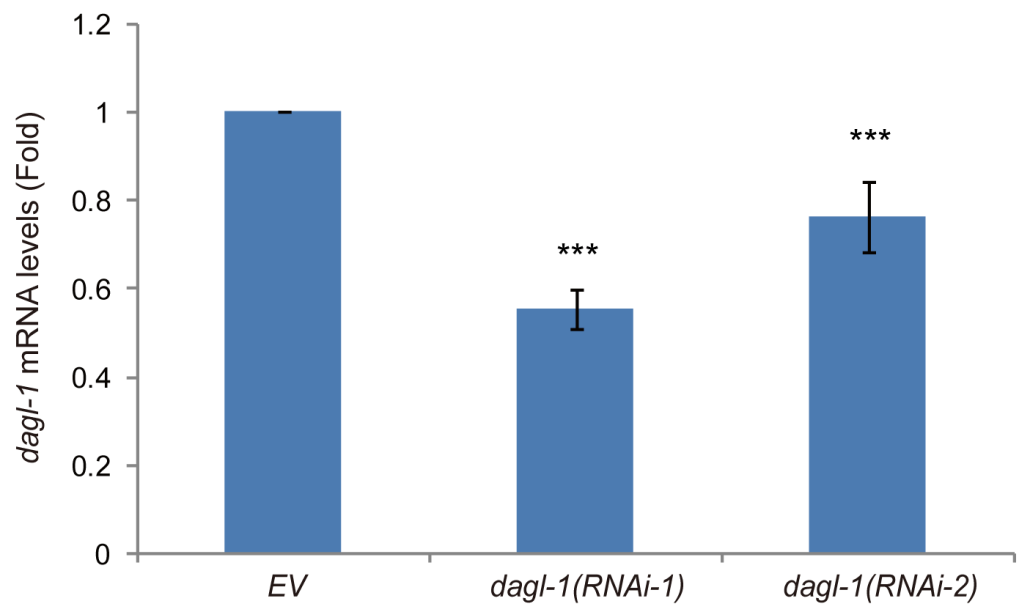


Fig. S5. Real-time quantitative PCR analysis shows reduced *dagl-1* expression upon RNAi knockdown in N2 worms. N2 worms treated with bacteria harboring an RNAi construct targeting either 5’ (*dagl-1(RNAi-1)*) or 3’ (*dagl-1(RNAi-2)*) mRNA of *dagl-1* show a reduction of 45% and 24% in *dagl-1* transcript levels compared to that of the control treated with bacteria containing only empty vector (N2 *EV*). Fold-change of *dagl-1* expression is normalized to the levels of the housekeeping gene, *actin*, and shown relative to N2 *EV*.


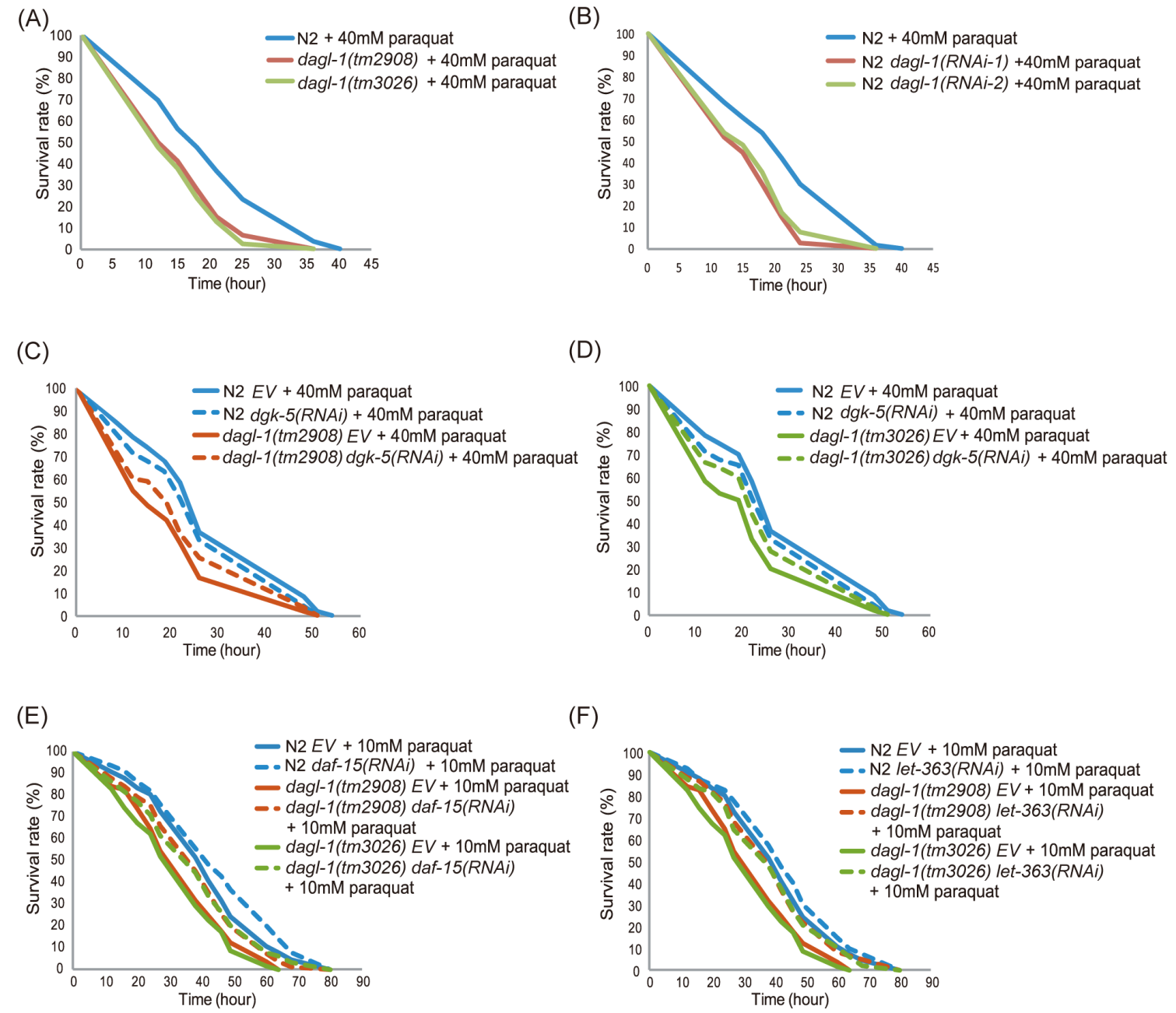


Fig. S6. *C. elegans* *dagl-1* mutants show reduced tolerance to oxidative stress that can be rescued by RNAi knockdown of *dgk-5*, *daf-15*, or *let-363*. (A) *dagl-1* mutants, *dagl-1(tm2908)* and *dagl-1(tm3026)*, show a reduction in mean survival of 21% (*P* < 0.001) and 26% (*P* < 0.001), respectively, compared to control N2 under paraquat-induced oxidative stress. (B) RNAi knockdown of *dagl-1* by *dagl-1(RNAi-1)* or *dagl-1(RNAi-2)* decreases survival by 22% (*P* < 0.001) or 27% (*P* < 0.001), respectively, compared to control N2 under paraquat-induced oxidative stress. (C, D, E, F) Reduced tolerance to paraquat-induced oxidative stress in *dagl-1(tm2908)* and *dagl-1(tm3026)* can be rescued by RNAi knockdown of *dgk-5*, *daf-15*, or *let-363*. See also Table S4.


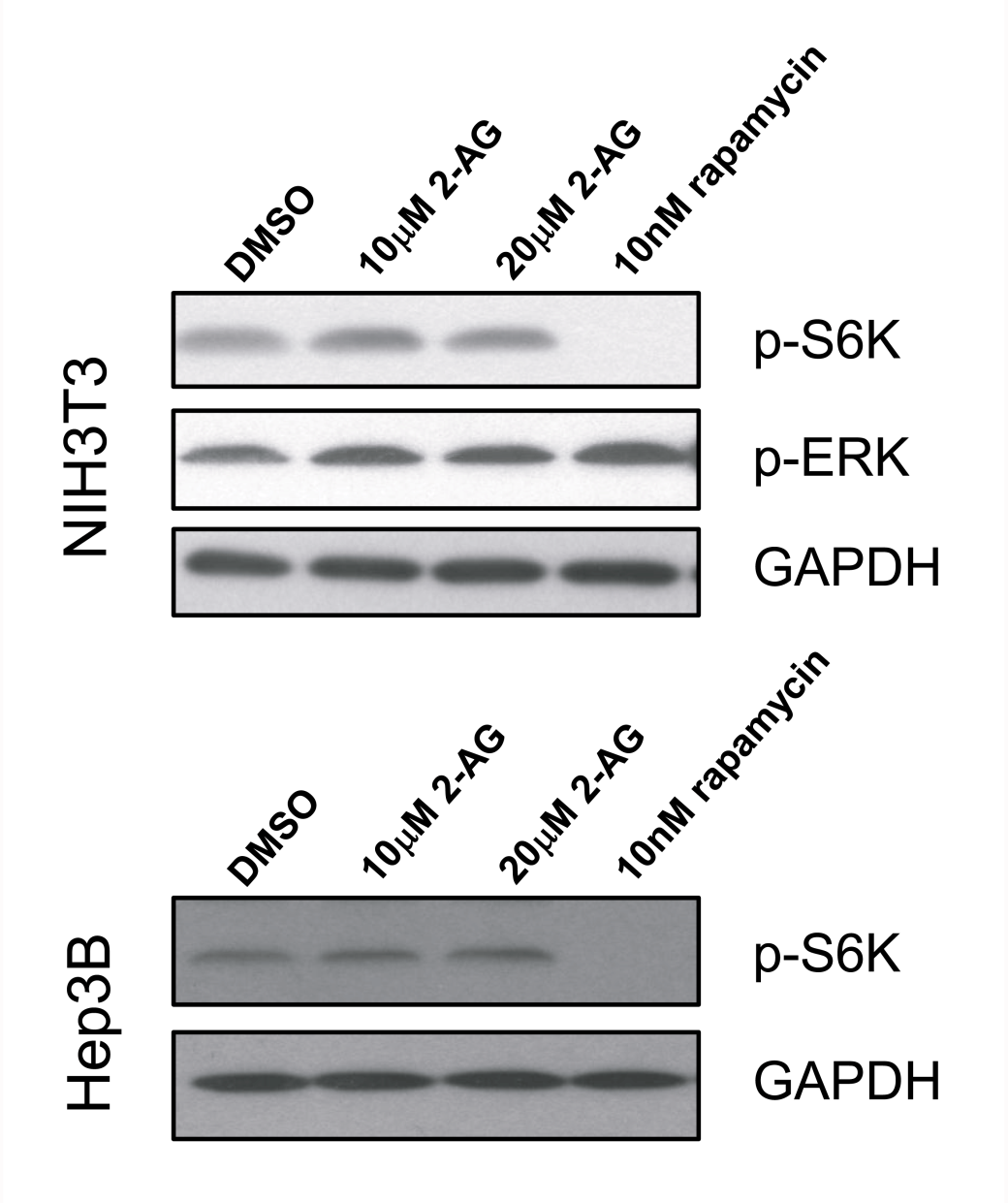


Fig. S7. Exogenous supplementation of 2-AG does not reduce levels of phosphorylated-S6K (p-S6K) in NIH3T3 and Hep3B cell lines. To exclude the possibility that 2-AG can directly reduce levels of p-S6K, NIH3T3 (upper panel) and Hep3B (lower panel) cells were treated with 10 µM or 20 µM 2-AG, or 10 nM of rapamycin, for 24 h and then harvested for western blot analysis. Treatment with 2-AG increases levels of p-ERK, consistent with previous reports ([Zhao *et al.* 2005](#_ENREF_1)). However, neither concentration of 2-AG affects levels of p-S6K compared to that of cells mock-treated with DMSO. In contrast, rapamycin treatment significantly decreased p-S6K levels in both cell lines. GAPDH was used as an internal control.


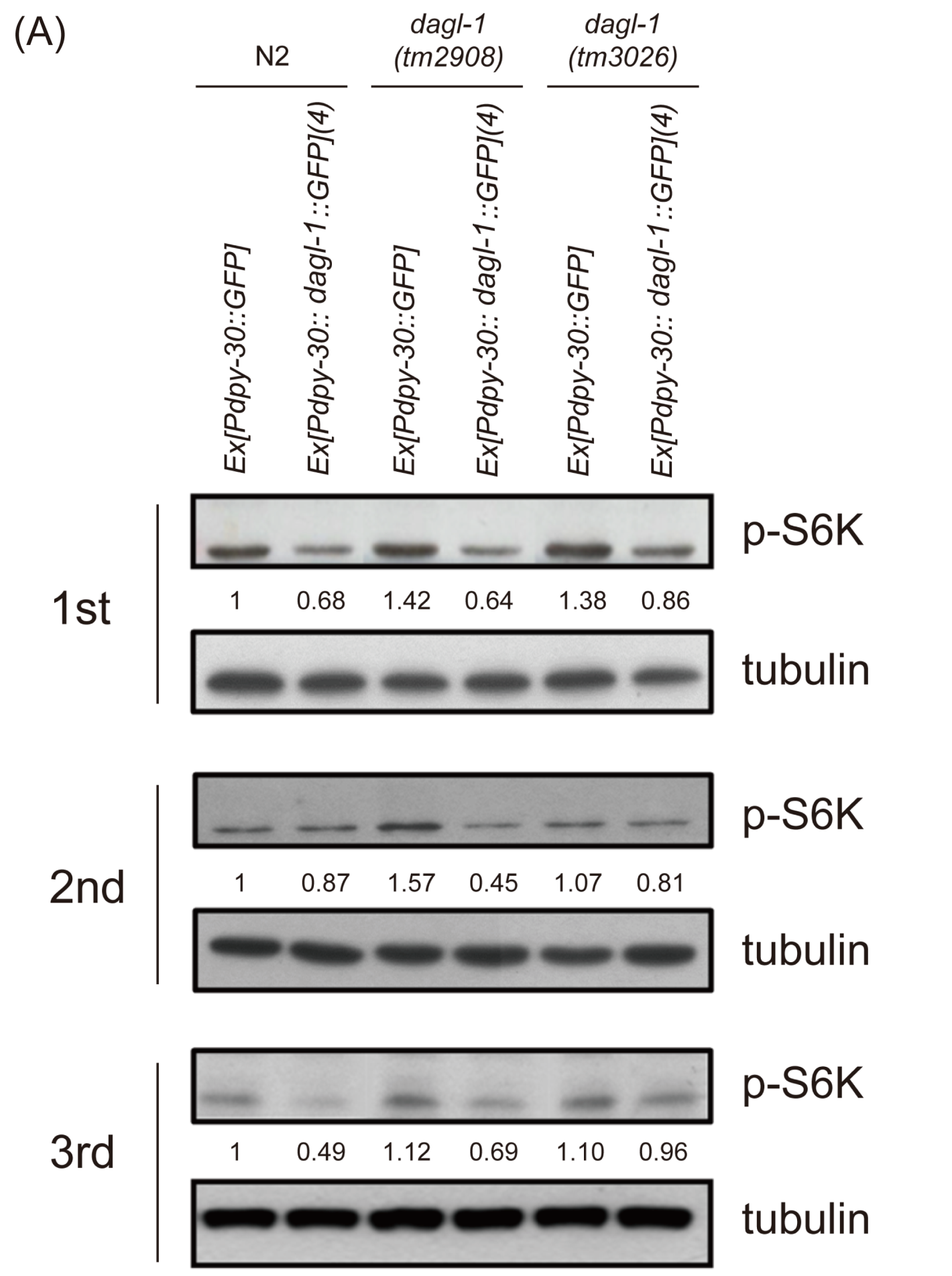


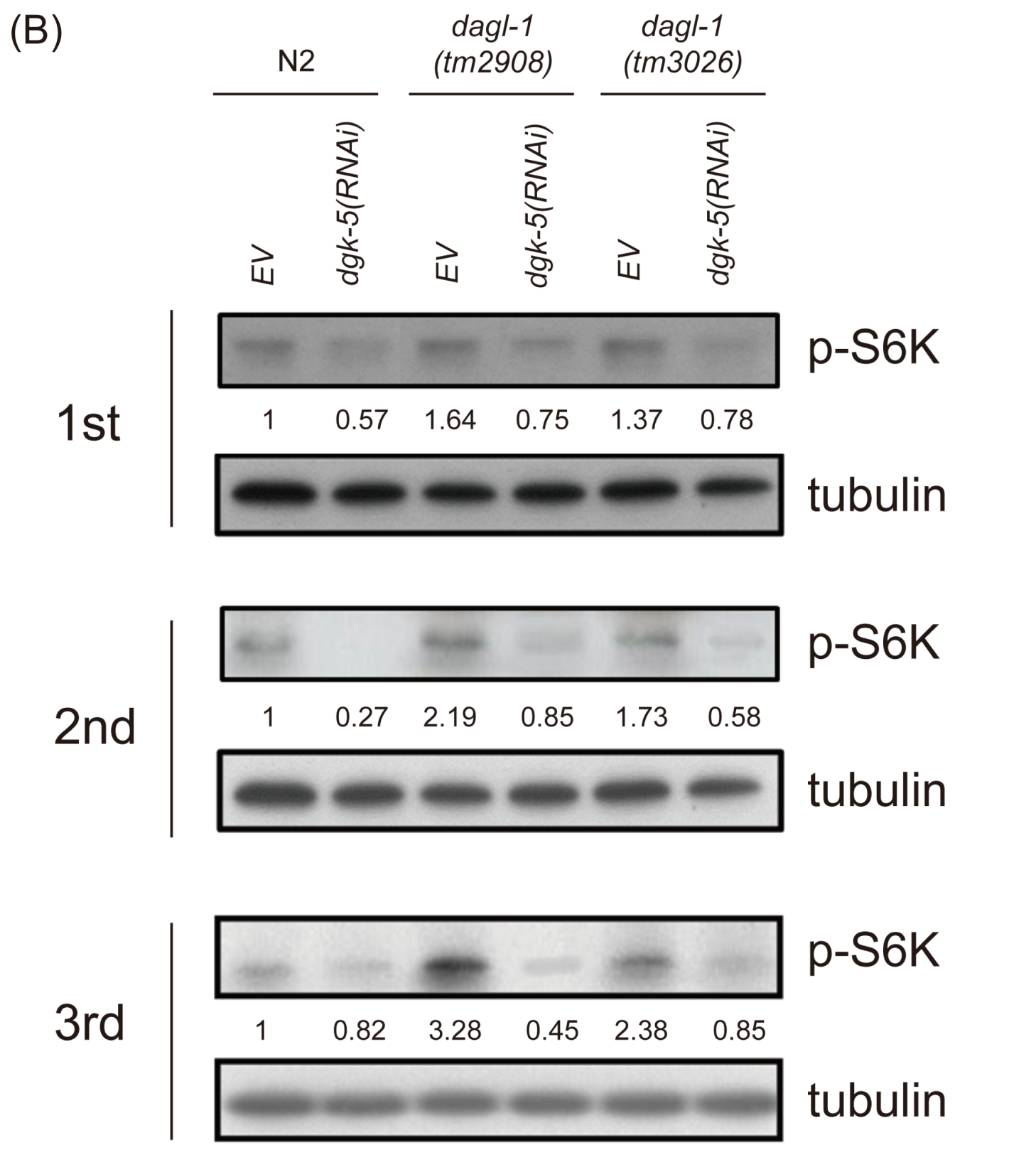


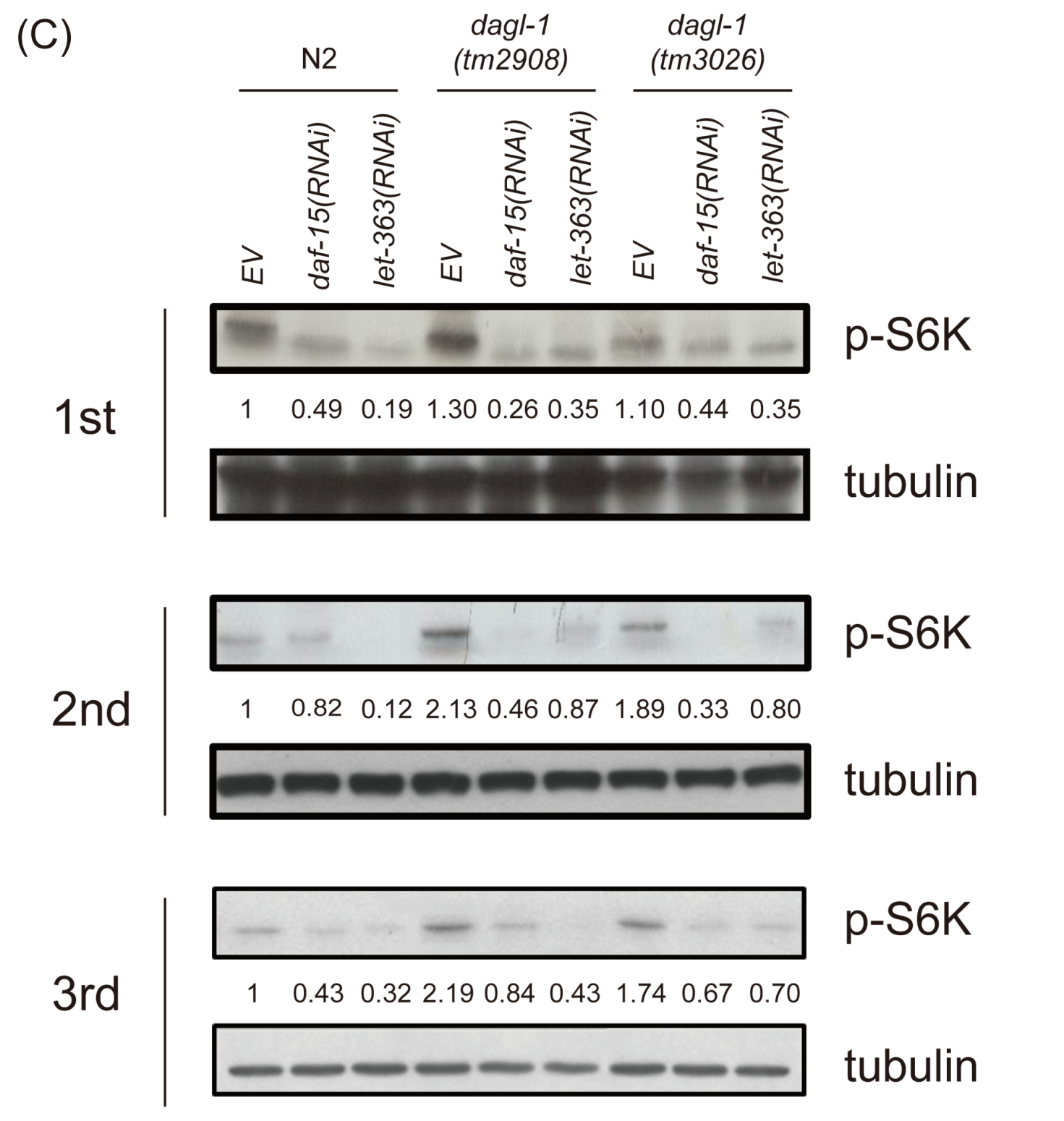


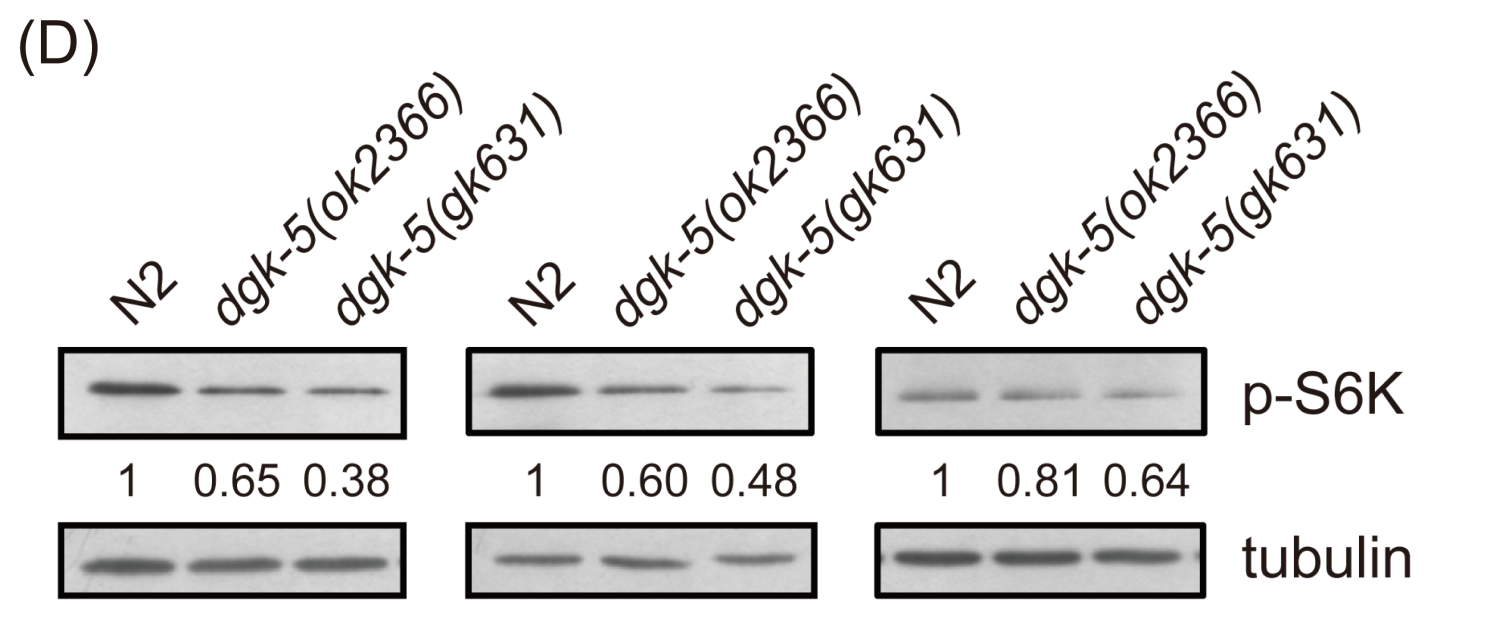


Fig. S8. Western blots for p-S6K. Independent experiments are shown for the results in Figs. (A) 3H, (B) 4A, (C) 4C, and (D) S3E. Fold-changes are listed below the blots and measured as described in the Experimental Procedures.

**
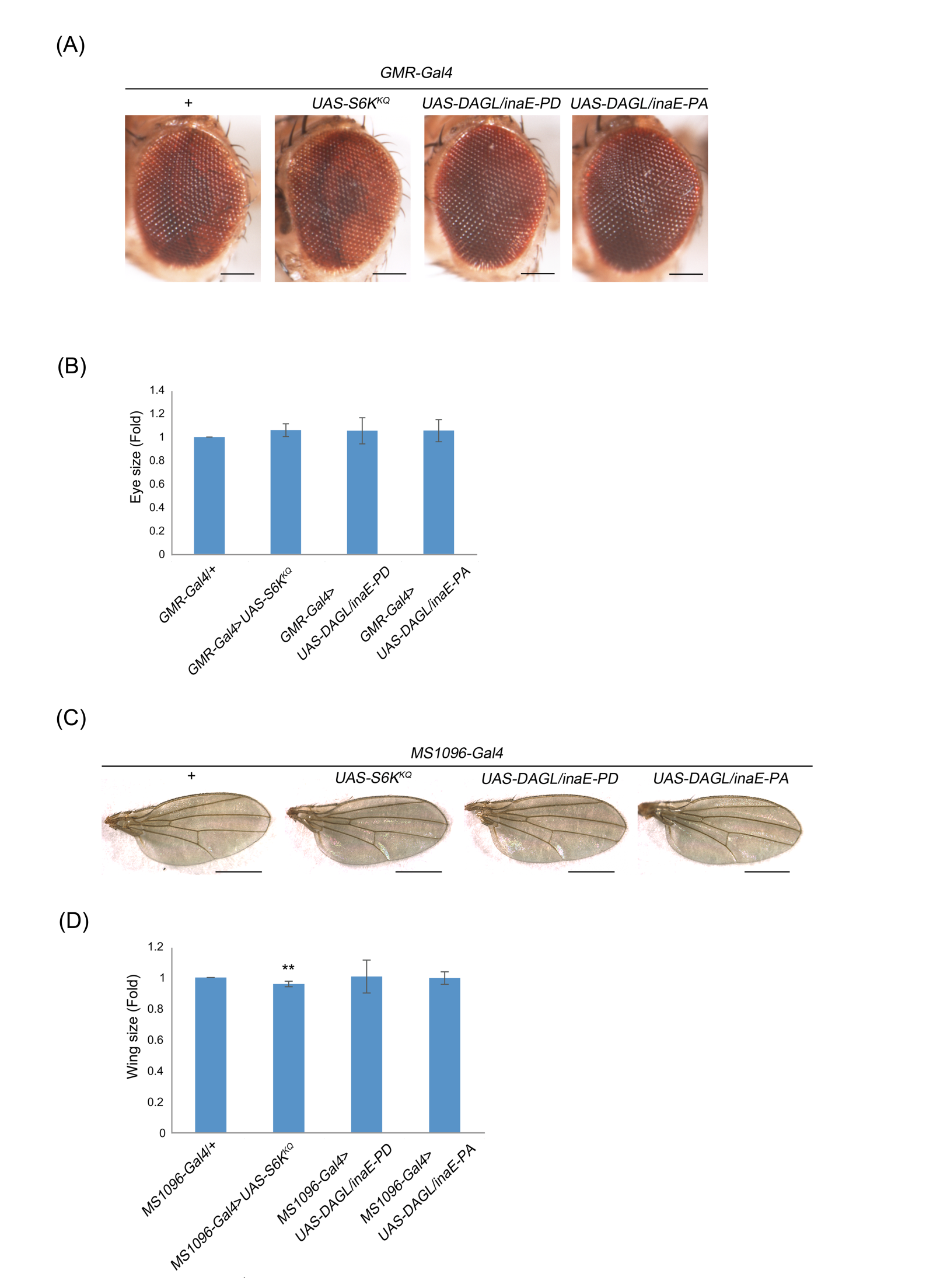
**

Fig. S9. No smaller eye or wing size was detected upon *DAGL*/*inaE* overexpression. (A) No differences of eye sizes were observed in the *Drosophila* eye by the overexpression of either *DAGL*/*inaE-PD* or *DAGL*/*inaE-PA* compared to the control *GMR-Gal4/+*. The bar represents 100μm. (B) No changes were detected in the eye sizes among the different alleles (n=10 for each allele). The eye size was circled and measured by the software NIS-Elements D of Nikon dissecting microscope SMZ1500. The eye size of *GMR-Gal4/+* was used as the standard for normalization. (C) No differences of wing sizes were observed in the *Drosophila* wing by the overexpression of either *DAGL*/*inaE-PD* or *DAGL*/*inaE-PA* compared to the control *MS1096-Gal4/+*. The bar represents 500μm. (D) No changes were detected in the wing sizes by overexpression of either *DAGL*/*inaE-PD* or *DAGL*/*inaE-PA* (n=7 for each allele). The wing size was circled and measured by the software NIS-Elements D. The wing size of *MS1096-Gal4/+* was used as the standard for normalization.

| Table S1. Lifespan of *DAGL*/*inaE* transgenic overexpression flies by different Gal4 drivers. | | | | | | |
| --- | --- | --- | --- | --- | --- | --- |
| Strain | Mean Lifespan (Days) | Difference (%) | | p-value | n | |
| *hs-GAL4/+* | 48 | - | | - | 66 |  |
| *UAS-DAGL*/*inaE-PD/+* | 45 | - | | - | 41 |  |
| *hs-GAL4>UAS-*  *DAGL*/*inaE-PD* | 59 | 30.9 ^a^, 23.5 ^b^ | | <0.001^a,b^ *** | 55 |  |
| *UAS-DAGL*/*inaE-PA/+* | 46 | - | | - | 46 |  |
| *hs-GAL4>UAS- DAGL*/*inaE-PA* | 57 | 23.6 ^a^, 18.8 ^c^ | | <0.001^a,c^ *** | 32 |  |
| *da-GAL4/+* | 52 | - | | - | 45 |  |
| *UAS-DAGL*/*inaE-PD/+* | 45 | - | | - | 46 |  |
| *da-GAL4>UAS- DAGL*/*inaE-PD* | 62 | 36.7 ^d^, 18.5 ^b^ | | <0.001^d^***, <0.05^b^* | 45 |  |
| *UAS-DAGL*/*inaE-PA/+* | 46 | - | | - | 41 |  |
| *da-GAL4>UAS- DAGL*/*inaE-PA* | 62 | 34.4 ^d^, 18.5 ^c^ | | <0.001^d^*** , <0.05^c^* | 47 |  |
| *GMR-GAL4/+* | 44 | - | | - | 67 |  |
| *UAS-DAGL*/*inaE-PD/+* | 45 | - | | - | 41 |  |
| *GMR-GAL4>UAS- DAGL*/*inaE-PD* | 58 | 32.2 ^e^, 29.5 ^b^ | | <0.001^e,b^ *** | 69 |  |
| *UAS-DAGL*/*inaE-PA/+* | 46 | - | | - | 46 |  |
| *GMR-GAL4>UAS- DAGL*/*inaE-PA* | 51 | 15.9 ^e^, 12.3 ^c^ | | <0.05^e,c^* | 57 |  |
| *Appl-GAL4/+* | 51 | - | | - | 49 |  |
| *UAS-DAGL*/*inaE-PD/+* | 45 | - | | - | 41 |  |
| *Appl-GAL4>UAS- DAGL*/*inaE-PD* | 57 | 10.0 ^f^, 25.8 ^b^ | | <0.05^f^* , <0.001^b^*** | 47 |  |
| *UAS-DAGL*/*inaE-PA/+* | 46 | - | | - | 46 |  |
| *Appl-GAL4>UAS- DAGL*/*inaE-PA* | 63 | 21.9 ^f^, 37.0 ^c^ | | <0.001^f,c^ *** | 39 |  |
| Lifespan is carried out at 25°C. *P* values are calculated by log rank test. (**P*<0.05, ****P*<0.001) | | | | | | |
| ^a^ Compared to *hs-GAL4/+*. | | | ^b^ Compared to *UAS-DAGL*/*inaE-PD/+*. | | | |
| ^c^ Compared to *UAS-DAGL*/*inaE-PA/+*. | | | ^d^ Compared to *da-GAL4/+*. | | | |
| ^e^ Compared to *GMR-GAL4/+*. | | | ^f^ Compared to *Appl-GAL4/+*. | | | |

| Table S2. Oxidative stress^g^ response of *DAGL*/*inaE* transgenic overexpression flies by different Gal4 drivers. | | | | | | |
| --- | --- | --- | --- | --- | --- | --- |
| Strain | Mean Lifespan (Hours) | Difference (%) | | p-value | n | |
| *hs-GAL4/+* | 29 | - | | - | 135 |  |
| *UAS-DAGL*/*inaE-PD/+* | 37 | - | | - | 231 |  |
| *hs-GAL4>UAS- DAGL*/*inaE-PD* | 43 | 48.2^a^, 18.6^b^ | | <0.001^a^***, <0.05^b^* | 149 |  |
| *UAS-DAGL*/*inaE-PA/+* | 34 | - | | - | 187 |  |
| *hs-GAL4>UAS- DAGL*/*inaE-PA* | 44 | 50.9^a^, 29.2^c^ | | <0.001^a^***, <0.05^c^* | 236 |  |
| *da-GAL4/+* | 44 | - | | - | 112 |  |
| *UAS-DAGL*/*inaE-PD/+* | 37 | - | | - | 231 |  |
| *da-GAL4>UAS- DAGL*/*inaE-PD* | 49 | 11.9^d^, 33.9^b^ | | <0.05^d^*, <0.001^b^*** | 176 |  |
| *UAS-DAGL*/*inaE-PA/+* | 34 | - | | - | 187 |  |
| *da-GAL4>UAS- DAGL*/*inaE-PA* | 47 | 7.3^d^, 37.4^c^ | | <0.05^d, c^* | 107 |  |
| *GMR-GAL4/+* | 63 | - | | - | 124 |  |
| *UAS-DAGL*/*inaE-PD/+* | 36 | - | | - | 231 |  |
| *GMR-GAL4>UAS- DAGL*/*inaE-PD* | 96 | 54.2^e^,169.3^b^ | | <0.05^e^* , <0.001^b^*** | 195 |  |
| *UAS-DAGL*/*inaE-PA/+* | 34 | - | | - | 187 |  |
| *GMR-GAL4>UAS- DAGL*/*inaE-PA* | 70 | 11.7^e^, 104.1^c^ | | <0.05^e^* , <0.001^c^*** | 167 |  |
| *Appl-GAL4/+* | 51 | - | | - | 61 |  |
| *UAS-DAGL*/*inaE-PD/+* | 36 | - | | - | 231 |  |
| *Appl-GAL4>UAS- DAGL*/*inaE-PD* | 84 | 64.4^f^, 133.2^b^ | | <0.05^f^* , <0.001^b^*** | 236 |  |
| *UAS-DAGL*/*inaE-PA/+* | 34 | - | | - | 187 |  |
| *Appl-GAL4>UAS- DAGL*/*inaE-PA* | 64 | 26.8^f^, 88.3^c^ | | <0.05^f^* , <0.001^c^*** | 144 |  |
| *P* values are calculated by Student’s t-test. (**P*<0.05, ****P*<0.001) | | | | | | |
| ^a^ Compared to *hs-GAL4/+*. | | | ^b^ Compared to *UAS-DAGL*/*inaE-PD/+*. | | | |
| ^c^ Compared to *UAS-DAGL*/*inaE-PA/+*. | | | ^d^ Compared to *da-GAL4/+*. | | | |
| ^e^ Compared to *GMR-GAL4/+*. | | | ^f^ Compared to *Appl-GAL4/+*. | | | |
| ^g^ 20mM paraquat in 5% sucrose water. | | |  | | | |

| Table S3^d^. Effect of *dagl-1* expression on lifespan of N2 and *dagl-1(tm2908)* and *dagl-1(tm3026)* in *C. elegans*. | | | | |
| --- | --- | --- | --- | --- |
| Strain | Mean Lifespan ±SEM(Days) | Difference (%) | p-value | n |
| N2*; Ex[Pdpy-30::GFP]* | 13.5±0.2 | - | - | 121 |
| N2*; Ex[Pdpy-30::dagl-1::GFP](3)* | 15.1±0.7 | 12.17 | 0.002^a^** | 104 |
| N2*; Ex[Pdpy-30::dagl-1::GFP](4)* | 15.2±0.3 | 12.76 | <0.001^a^*** | 84 |
| N2 *EV* | 12.8±0.7 | - | - | 119 |
| N2 *dagl-1(RNAi-1)* | 11.1±0.5 | -13.65 ^b^ | <0.001^b^*** | 115 |
| N2 *dagl-1(RNAi-2)* | 11.3±0.3 | -11.70 ^b^ | <0.001^b^*** | 116 |
| N2 | 12.8±0.9 | - | - | 235 |
| *dagl-1(tm2908)* | 10.2±1.0 | -19.65 ^c^ | <0.001^c^*** | 217 |
| N2 | 13.9±1.0 | - | - | 120 |
| *dagl-1(tm3026)* | 12.1±1.0 | -13.06 ^c^ | 0.004^c^** | 123 |
| *N2; Ex[Pdpy-30::GFP]* | 13.0±0.3 | - | - | 108 |
| *N2; Ex[Pdpy-30::dagl-1::GFP](4)* | 14.2±0.8 | 9.81 ^a^ | 0.035^a^* | 77 |
| *dagl-1(tm2908); Ex[Pdpy-30::GFP]* | 11.8±0.2 | -8.96 ^a^ | 0.005^a^** | 93 |
| *dagl-1(tm2908); Ex[Pdpy-30::dagl-1::GFP](4)* | 12.5±0.2 | -3.32 ^a^ | 0.024^a^* | 79 |
| *dagl-1(tm3026); Ex[Pdpy-30::GFP]* | 11.8±0.3 | -9.03 ^a^ | <0.001^a^*** | 78 |
| *dagl-1(tm3026); Ex[Pdpy-30::dagl-1::GFP](4)* | 12.1±0.4 | -6.33 ^a^ | 0.004 ^a^** | 75 |
| Lifespan is carried out at 20°C. *P* values are calculated by log rank test. (**P*<0.05, ***P*<0.01, ****P*<0.001)  ^a^ Compared to N2*; Ex[Pdpy-30::GFP]* grown on *E. coli OP50*.  ^b^ Compared to N2 grown on empty vector control.  ^c^ Compared to N2 grown on *E. coli OP50*.  ^d^ The three separate data were listed in Table S7. | | | | |

| Table S4 ^d^. Effect of *dagl-1* expression and knockdown of *dgk-5, let-363,* *daf-15* on oxidative stress^c^ response of N2 and *dagl-1(tm2908)* and *dagl-1(tm3026)* in *C. elegans*. | | | | | | | | | | | |  |
| --- | --- | --- | --- | --- | --- | --- | --- | --- | --- | --- | --- | --- |
|  | | Strain | Mean Lifespan ±SEM(hours) | | | Difference (%) | | p-value | | n | | |
| 40mM paraquat^c^ | N2 | | | 21.5±0.3 | - | | - | | 96 | | | |
|  | *dagl-1(tm2908)* | | | 16.9±0.3 | -21.34^a^ | | <0.001^a^*** | | 93 | | | |
|  | *dagl-1(tm3026)* | | | 16.0±0.5 | -25.85^a^ | | <0.001^a^*** | | 87 | | | |
| 40mM paraquat | N2 *EV* | | | 21.4±0.4 | - | | - | | 76 | | | |
|  | N2 *dagl-1(RNAi-1)* | | | 16.6±0.2 | -22.29 | | <0.001^b^*** | | 68 | | | |
|  | N2 *dagl-1(RNAi-2)* | | | 15.7±0.6 | -26.87 | | <0.001^b^*** | | 60 | | | |
| 40mM  Paraquat | N2 *EV* | | | 24.3±2.9 | - | | - | | 58 | | | |
|  | N2 *dgk-5(RNAi)* | | | 27.8±1.0 | 14.73 | | 0.020^b^* | | 54 | | | |
|  | *dagl-1(tm2908) EV* | | | 16.0±1.6 | -34.16 | | 0.002^b^** | | 52 | | | |
|  | *dagl-1(tm2908) dgk-5(RNAi)* | | | 22.3±2.9 | -7.96 | | 0.321^b^ | | 59 | | | |
|  | *dagl-1(tm3026) EV* | | | 17.3±1.5 | -28.59 | | 0.005^b^** | | 58 | | | |
|  | *dagl-1(tm3026) dgk-5(RNAi)* | | | 25.6±1.3 | 5.38 | | 0.343^b^ | | 55 | | | |
| 10mM  Paraquat | N2 *EV* | | | 41.5±2.4 | - | | - | | 66 | | | |
|  | N2 *let-363(RNAi)* | | | 44.2±1.6 | 6.54 | | 0.577^b^ | | 40 | | | |
|  | N2 *daf-15(RNAi)* | | | 45.4±1.7 | 9.48 | | 0.361^b^ | | 79 | | | |
|  | *dagl-1(tm2908) EV* | | | 33.8±3.1 | -18.50 | | 0.047^b^* | | 57 | | | |
|  | *dagl-1(tm2908) let-363(RNAi)* | | | 39.9±1.9 | -3.90 | | 0.578^b^ | | 60 | | | |
|  | *dagl-1(tm2908) daf-15(RNAi)* | | | 38.3±2.0 | -7.72 | | 0.390^b^ | | 82 | | | |
|  | *dagl-1(tm3026) EV* | | | 32.1±2.2 | -22.57 | | 0.012^b^* | | 58 | | | |
|  | *dagl-1(tm3026) let-363(RNAi)* | | | 38.9±1.7 | -6.29 | | 0.498^b^ | | 48 | | | |
|  | *dagl-1(tm3026) daf-15(RNAi)* | | | 37.7±1.5 | -9.06 | | 0.219^b^ | | 65 | | | |
| Oxidative stress resistance experiments are carried out at 20°C. *P* values are calculated by Student’s t-test. (**P*<0.05, ***P*<0.01, ****P*<0.001)  ^a^ Compared to N2 grown on *E. coli OP50*.  ^b^ Compared to N2 grown on empty vector control.  ^c^ Specific paraquat concentration was prepared in S-medium.  ^d^ The three separate data were listed in Table S8. | | | | | | | | | | |  |  |

| Table S5 ^b^. Effect of *dgk-5, let-363,* and *daf-15* RNAi knockdown on the lifespan of N2 and *dagl-1(tm2908)* and *dagl-1(tm3026)* in *C. elegans*. | | | | | |
| --- | --- | --- | --- | --- | --- |
| Strain | Mean Lifespan ±SEM(Days) | Difference (%) | p-value | n | |
| N2 *EV* | 12.6±0.2 | - | - | 377 | |
| N2 *dgk-5(RNAi)* | 14.0±0.4 | 10.75 | <0.001^a^*** | 325 | |
| *dagl-1(tm2908) EV* | 10.0±0.2 | -20.86 | <0.001^a^*** | 239 | |
| *dagl-1(tm2908) dgk-5(RNAi)* | 12.7±0.6 | 0.65 | 0.393 ^a^ | 301 | |
| *dagl-1(tm3026) EV* | 10.3±0.3 | -18.61 | <0.001^a^*** | 238 | |
| *dagl-1(tm3026) dgk-5(RNAi)* | 12.7±0.3 | 0.80 | <0.105 ^a^ | 228 | |
| N2 *EV* | 13.0±0.4 |  | - | 164 | |
| N2 *let-363(RNAi)* | 14.3±0.4 | 10.07 | <0.001^a^*** | 86 | |
| *dagl-1(tm2908) EV* | 10.8±0.3 | -17.02 | <0.001^a^*** | 104 | |
| *dagl-1(tm2908) let-363(RNAi)* | 14.1±0.4 | 7.89 | 0.002 ^a^** | 77 | |
| *dagl-1(tm3026) EV* | 11.4±0.3 | -12.24 | <0.001^a^*** | 93 | |
| *dagl-1(tm3026) let-363(RNAi)* | 14.1±0.4 | 8.29 | <0.001^a^*** | 94 | |
| N2 *EV* | 13.0±0.4 |  | - | 164 | |
| N2 *daf-15(RNAi)* | 14.4±0.4 | 10.09 | <0.001^a^*** | 91 | |
| *dagl-1(tm2908) EV* | 10.8±0.3 | -17.02 | <0.001^a^*** | 104 | |
| *dagl-1(tm2908) daf-15(RNAi)* | 13.8±0.3 | 6.17 | 0.008 ^a^** | 94 | |
| *dagl-1(tm3026) EV* | 11.4±0.3 | -12.24 | <0.001^a^*** | 93 | |
| *dagl-1(tm3026) daf-15(RNAi)* | 14.0±0.3 | 7.39 | 0.01 ^a^* | 84 | |
| Lifespan is carried out at 20°C. *P* values are calculated by log rank test. (**P*<0.05, ***P*<0.01, ****P*<0.001)  ^a^ Compared to N2 grown on empty vector control.  ^b^ The three separate data were listed in Table S9. | | | | |  |

| Table S6 ^b^. The lifespan of N2, *dgk-5(ok2366)* and *dgk-5(gk631)* in *C. elegans*. | | | | |
| --- | --- | --- | --- | --- |
| Strain | Mean Lifespan ±SEM(Days) | Difference (%) | p-value | n |
| N2 | 12.3±0.3 | - | - | 216 |
| *dgk-5(ok2366)* | 13.3±0.3 | 7.99 | 0.029 ^a^ * | 287 |
| *dgk-5(gk631)* | 13.6±0.4 | 10.84 | 0.004 ^a^ ** | 207 |

| Lifespan is carried out at 20°C. *P* values are calculated by log rank test. (**p*<0.05, ***p*<0.01, ****p*<0.001)  ^a^ Compared to N2 grown on *E. coli OP50*.  ^b^ The three separate data were listed in Table S10. |
| --- |

| Table S7. Effect of *dagl-1* expression on lifespan of N2 and *dagl-1(tm2908)* and *dagl-1(tm3026)* in *C. elegans*. (the three separate data for Table S3) | | | | |
| --- | --- | --- | --- | --- |
| Strain | Mean Lifespan (Days) | Difference (%) | *P*-value | n |
| N2*; Ex[Pdpy-30::GFP]* | 13.4 | - | - | 42 |
| N2*; Ex[Pdpy-30::dagl-1::GFP](3)* | 14.7 | 8.78 | 0.047 ^a^* | 30 |
| N2*; Ex[Pdpy-30::dagl-1::GFP](4)* | 14.7 | 9.22 | 0.037 ^a^ * | 42 |
| N2*; Ex[Pdpy-30::GFP]* | 14.0 | - | - | 33 |
| N2*; Ex[Pdpy-30::dagl-1::GFP](3)* | 15.1 | 7.39 | <0.001 ^a^ *** | 30 |
| N2*; Ex[Pdpy-30::dagl-1::GFP](4)* | 15.8 | 12.51 | 0.048 ^a^ * | 14 |
| N2*; Ex[Pdpy-30::GFP]* | 13.6 | - | - | 46 |
| N2*; Ex[Pdpy-30::dagl-1::GFP](3)* | 15.3 | 12.98 | 0.043 ^a^ * | 44 |
| N2*; Ex[Pdpy-30::dagl-1::GFP](4)* | 15.5 | 14.26 | <0.001 ^a^ *** | 28 |
| N2 *EV* | 12.9 | - | - | 40 |
| N2 *dagl-1(RNAi-1)* | 11.1 | -13.62 | 0.007 ^b^ ** | 38 |
| N2 *dagl-1(RNAi-2)* | 11.3 | -11.87 | 0.021 ^b^ * | 39 |
| N2 *EV* | 12.7 | - | - | 40 |
| N2 *dagl-1(RNAi-1)* | 11.2 | -11.82 | 0.035 ^b^ * | 38 |
| N2 *dagl-1(RNAi-2)* | 11.2 | -11.81 | 0.043 ^b^ * | 45 |
| N2 *EV* | 13.1 | - | - | 39 |
| N2 *dagl-1(RNAi-1)* | 11.0 | -16.38 | <0.001 ^b^ *** | 39 |
| N2 *dagl-1(RNAi-2)* | 11.4 | -12.98 | <0.001 ^b^ *** | 32 |
| N2 | 12.3 | - | - | 67 |
| *dagl-1(tm2908)* | 10.5 | -14.05 | <0.001 ^c^ *** | 62 |
| N2 | 12.3 | - | - | 88 |
| *dagl-1(tm2908)* | 10.2 | -16.91 | <0.001 ^c^ *** | 67 |
| N2 | 13.7 | - | - | 80 |
| *dagl-1(tm2908)* | 10.1 | -26.41 | <0.001 ^c^ *** | 88 |
| N2 | 12.7 | - | - | 34 |
| *dagl-1(tm3026)* | 11.2 | -12.41 | 0.048 ^c^ * | 58 |
| N2 | 14.2 | - | - | 42 |
| *dagl-1(tm3026)* | 12.9 | -8.82 | 0.003 ^c^ ** | 49 |
| N2 | 14.5 | - | - | 68 |
| *dagl-1(tm3026)* | 12.7 | -12.16 | 0.023 ^c^ * | 38 |
| *N2; Ex[Pdpy-30::GFP]* | 12.9 | - | - | 30 |
| *N2; Ex[Pdpy-30::dagl-1::GFP](4)* | 13.9 | 7.79 | 0.05 ^a^* | 22 |
| *dagl-1(tm2908); Ex[Pdpy-30::GFP]* | 11.6 | -10.35 | <0.001 ^a^ *** | 23 |
| *dagl-1(tm2908); Ex[Pdpy-30::dagl-1::GFP](4)* | 12.4 | -3.71 | 0.176 ^a^ | 19 |
| *dagl-1(tm3026); Ex[Pdpy-30::GFP]* | 11.3 | -12.79 | <0.001 ^a^ *** | 20 |
| *dagl-1(tm3026); Ex[Pdpy-30::dagl-1::GFP](4)* | 12.4 | -3.96 | 0.242 ^a^ | 18 |
| *N2; Ex[Pdpy-30::GFP]* | 13.1 | - | - | 28 |
| *N2; Ex[Pdpy-30::dagl-1::GFP](4)* | 13.9 | 6.28 | 0.015 ^a^ * | 26 |
| *dagl-1(tm2908); Ex[Pdpy-30::GFP]* | 11.7 | -10.75 | 0.018 ^a^ * | 27 |
| *dagl-1(tm2908); Ex[Pdpy-30::dagl-1::GFP](4)* | 12.5 | -4.75 | 0.442 ^a^ | 20 |
| *dagl-1(tm3026); Ex[Pdpy-30::GFP]* | 11.4 | -12.72 | 0.009 ^a^ ** | 22 |
| *dagl-1(tm3026); Ex[Pdpy-30::dagl-1::GFP](4)* | 12.2 | -6.81 | 0.352 ^a^ | 22 |
| *N2; Ex[Pdpy-30::GFP]* | 13.0 | - | - | 50 |
| *N2; Ex[Pdpy-30::dagl-1::GFP](4)* | 14.9 | 13.94 | 0.001 ^a^ ** | 28 |
| *dagl-1(tm2908); Ex[Pdpy-30::GFP]* | 12.0 | -8.33 | <0.001 ^a^ *** | 43 |
| *dagl-1(tm2908); Ex[Pdpy-30::dagl-1::GFP](4)* | 12.7 | -2.42 | 0.302 ^a^ | 40 |
| *dagl-1(tm3026); Ex[Pdpy-30::GFP]* | 12.1 | -7.12 | 0.004 ^a^ ** | 36 |
| *dagl-1(tm3026); Ex[Pdpy-30::dagl-1::GFP](4)* | 13.0 | -0.31 | 0.955 ^a^ | 35 |

| Lifespan is carried out at 20°C. *P* values are calculated by log rank test. (**P*<0.05, ***P*<0.01, ****P*<0.001)  ^a^ Compared to N2*; Ex[Pdpy-30::GFP]* grown on *E. coli OP50*.  ^b^ Compared to N2 grown on empty vector control.  ^c^ Compared to N2 grown on *E. coli OP50*. |
| --- |

| Table S8. Effect of *dagl-1* expression and knockdown of *dgk-5, let-363,* *daf-15* on oxidative stress^c^ response of N2 and *dagl-1(tm2908)* and *dagl-1(tm3026)* in *C. elegans*. (the three separate data for Table S4) | | | | | | | | | | | |  |
| --- | --- | --- | --- | --- | --- | --- | --- | --- | --- | --- | --- | --- |
|  | | Strain | Mean Lifespan (hours) | | | Difference (%) | | *P*-value | | n | | |
| 40mM paraquat^c^ | N2 | | | 21.3 | - | | - | | 36 | | | |
|  | *dagl-1(tm2908)* | | | 16.4 | -23.00 | | <0.001^a^*** | | 32 | | | |
|  | *dagl-1(tm3026)* | | | 16.9 | -20.66 | | 0.002^a^** | | 29 | | | |
|  | N2 | | | 21.4 | - | | - | | 31 | | | |
|  | *dagl-1(tm2908)* | | | 16.7 | -21.96 | | 0.002^a^** | | 30 | | | |
|  | *dagl-1(tm3026)* | | | 16.0 | -25.23 | | <0.001^a^*** | | 30 | | | |
|  | N2 | | | 21.9 | - | | - | | 29 | | | |
|  | *dagl-1(tm2908)* | | | 16.9 | -22.83 | | <0.001^a^*** | | 31 | | | |
|  | *dagl-1(tm3026)* | | | 15.1 | -31.05 | | <0.001^a^*** | | 28 | | | |
| 40mM paraquat | N2 *EV* | | | 21.0 | - | | - | | 16 | | | |
|  | N2 *dagl-1(RNAi-1)* | | | 16.9 | -19.52 | | 0.002 ^b^ ** | | 14 | | | |
|  | N2 *dagl-1(RNAi-2)* | | | 15.8 | -24.76 | | <0.001 ^b^ *** | | 16 | | | |
|  | N2 *EV* | | | 21.9 | - | | - | | 31 | | | |
|  | N2 *dagl-1(RNAi-1)* | | | 16.3 | -25.57 | | <0.001 ^b^ *** | | 26 | | | |
|  | N2 *dagl-1(RNAi-2)* | | | 15.6 | -28.77 | | <0.001 ^b^ *** | | 14 | | | |
|  | N2 *EV* | | | 21.4 | - | | - | | 29 | | | |
|  | N2 *dagl-1(RNAi-1)* | | | 16.7 | -21.96 | | <0.001 ^b^ *** | | 28 | | | |
|  | N2 *dagl-1(RNAi-2)* | | | 15.7 | -26.64 | | <0.001 ^b^ *** | | 30 | | | |
| 40mM  Paraquat | N2 *EV* | | | 24.0 | - | | - | | 16 | | | |
|  | N2 *dgk-5(RNAi)* | | | 28.2 | 17.50 | | 0.027 ^b^ * | | 13 | | | |
|  | *dagl-1(tm2908) EV* | | | 16.0 | -33.33 | | 0.004 ^b^ ** | | 13 | | | |
|  | *dagl-1(tm2908) dgk-5(RNAi)* | | | 21.5 | -10.42 | | 0.124 ^b^ | | 14 | | | |
|  | *dagl-1(tm3026) EV* | | | 17.2 | -28.33 | | 0.006 ^b^ ** | | 14 | | | |
|  | *dagl-1(tm3026) dgk-5(RNAi)* | | | 25.1 | 4.58 | | 0.628 ^b^ | | 14 | | | |
|  | N2 *EV* | | | 25.0 | - | | - | | 14 | | | |
|  | N2 *dgk-5(RNAi)* | | | 27.5 | 10.0 | | 0.138 ^b^ | | 13 | | | |
|  | *dagl-1(tm2908) EV* | | | 16.1 | -35.60 | | 0.002 ^b^ ** | | 12 | | | |
|  | *dagl-1(tm2908) dgk-5(RNAi)* | | | 23.7 | -5.20 | | 0.374 ^b^ | | 15 | | | |
|  | *dagl-1(tm3026) EV* | | | 17.7 | -29.20 | | 0.005 ^b^ ** | | 14 | | | |
|  | *dagl-1(tm3026) dgk-5(RNAi)* | | | 25.5 | 2.00 | | 0.874 ^b^ | | 13 | | | |
|  | N2 *EV* | | | 24.1 | - | | - | | 28 | | | |
|  | N2 *dgk-5(RNAi)* | | | 27.8 | 15.35 | | 0.002 ^b^ ** | | 28 | | | |
|  | *dagl-1(tm2908) EV* | | | 16.0 | -33.61 | | <0.001 ^b^ *** | | 27 | | | |
|  | *dagl-1(tm2908) dgk-5(RNAi)* | | | 22.4 | -7.05 | | 0.062 ^b^ | | 30 | | | |
|  | *dagl-1(tm3026) EV* | | | 17.4 | -27.80 | | <0.001 ^b^ *** | | 30 | | | |
|  | *dagl-1(tm3026) dgk-5(RNAi)* | | | 25.9 | 7.47 | | 0.076 ^b^ | | 27 | | | |
| 10mM  Paraquat | N2 *EV* | | | 41.8 | - | | - | | 13 | | | |
|  | N2 *let-363(RNAi)* | | | 43.8 | 4.78 | | 0.307 ^b^ | | 11 | | | |
|  | N2 *daf-15(RNAi)* | | | 45.7 | 9.33 | | 0.183 ^b^ | | 19 | | | |
|  | *dagl-1(tm2908) EV* | | | 33.8 | -19.14 | | 0.082 ^b^ | | 13 | | | |
|  | *dagl-1(tm2908) let-363(RNAi)* | | | 39.8 | -4.78 | | 0.748 ^b^ | | 12 | | | |
|  | *dagl-1(tm2908) daf-15(RNAi)* | | | 38.4 | -8.13 | | 0.793 ^b^ | | 16 | | | |
|  | *dagl-1(tm3026) EV* | | | 31.9 | -23.68 | | 0.047 ^b^* | | 12 | | | |
|  | *dagl-1(tm3026) let-363(RNAi)* | | | 38.8 | -7.18 | | 0.862 ^b^ | | 11 | | | |
|  | *dagl-1(tm3026) daf-15(RNAi)* | | | 37.8 | -9.57 | | 0.524 ^b^ | | 20 | | | |
|  | N2 *EV* | | | 40.4 | - | | - | | 20 | | | |
|  | N2 *let-363(RNAi)* | | | 43.1 | 6.68 | | 0.865 ^b^ | | 9 | | | |
|  | N2 *daf-15(RNAi)* | | | 45.2 | 11.88 | | 0.217 ^b^ | | 20 | | | |
|  | *dagl-1(tm2908) EV* | | | 33.1 | -18.07 | | 0.071 ^b^ | | 15 | | | |
|  | *dagl-1(tm2908) let-363(RNAi)* | | | 39.2 | -2.97 | | 0.934 ^b^ | | 18 | | | |
|  | *dagl-1(tm2908) daf-15(RNAi)* | | | 48.0 | 18.81 | | 0.472 ^b^ | | 25 | | | |
|  | *dagl-1(tm3026) EV* | | | 31.8 | -21.29 | | 0.039 ^b^* | | 20 | | | |
|  | *dagl-1(tm3026) let-363(RNAi)* | | | 38.5 | -4.70 | | 0.574 ^b^ | | 22 | | | |
|  | *dagl-1(tm3026) daf-15(RNAi)* | | | 37.6 | -6.93 | | 0.711 ^b^ | | 16 | | | |
|  | N2 *EV* | | | 41.8 | - | | - | | 33 | | | |
|  | N2 *let-363(RNAi)* | | | 44.9 | 7.42 | | 0.445 ^b^ | | 20 | | | |
|  | N2 *daf-15(RNAi)* | | | 45.4 | 8.61 | | 0.240 ^b^ | | 40 | | | |
|  | *dagl-1(tm2908) EV* | | | 34.2 | -18.18 | | 0.017 ^b^* | | 29 | | | |
|  | *dagl-1(tm2908) let-363(RNAi)* | | | 40.3 | -3.59 | | 0.754 ^b^ | | 30 | | | |
|  | *dagl-1(tm2908) daf-15(RNAi)* | | | 38.4 | -8.13 | | 0.211 ^b^ | | 41 | | | |
|  | *dagl-1(tm3026) EV* | | | 32.5 | -22.25 | | 0.009 ^b^** | | 26 | | | |
|  | *dagl-1(tm3026) let-363(RNAi)* | | | 39.5 | -5.50 | | 0.921 ^b^ | | 15 | | | |
|  | *dagl-1(tm3026) daf-15(RNAi)* | | | 37.8 | -9.57 | | 0.181 ^b^ | | 29 | | | |
| Oxidative stress resistance experiments are carried out at 20°C. *P* values are calculated by Student’s t-test. (**P*<0.05, ***P*<0.01, ****P*<0.001)  ^a^ Compared to N2 grown on *E. coli OP50*.  ^b^ Compared to N2 grown on empty vector control.  ^c^ Specific paraquat concentration was prepared in S-medium. | | | | | | | | | | |  |  |

| Table S9. Effect of *dgk-5, let-363,* and *daf-15* RNAi knockdown on the lifespan of N2 and *dagl-1(tm2908)* and *dagl-1(tm3026)* in *C. elegans*. (the three separate data for Table S5) | | | | | |
| --- | --- | --- | --- | --- | --- |
| Strain | Mean Lifespan (Days) | Difference (%) | *P*-value | n | |
| N2 *EV* | 12.0 | - | - | 158 | |
| N2 *dgk-5(RNAi)* | 13.5 | 12.50 | <0.001^a^*** | 160 | |
| *dagl-1(tm2908) EV* | 9.8 | -18.33 | <0.001^a^*** | 78 | |
| *dagl-1(tm2908) dgk-5(RNAi)* | 11.9 | -0.82 | 0.04 ^a^* | 79 | |
| *dagl-1(tm3026) EV* | 9.9 | -17.51 | <0.001^a^*** | 71 | |
| *dagl-1(tm3026) dgk-5(RNAi)* | 12.1 | 0.83 | 0.21 ^a^ | 83 | |
| N2 *EV* | 13.1 | - | - | 88 | |
| N2 *dgk-5(RNAi)* | 14.9 | 13.74 | <0.001^a^*** | 66 | |
| *dagl-1(tm2908) EV* | 10.1 | -22.90 | <0.001^a^*** | 66 | |
| *dagl-1(tm2908) dgk-5(RNAi)* | 13.1 | 0.21 | 0.08 ^a^ | 126 | |
| *dagl-1(tm3026) EV* | 10.4 | -20.61 | <0.001^a^*** | 81 | |
| *dagl-1(tm3026) dgk-5(RNAi)* | 13.2 | 0.76 | 0.19 ^a^ | 77 | |
| N2 *EV* | 13.1 | - | - | 131 | |
| N2 *dgk-5(RNAi)* | 14.2 | 8.39 | 0.008^a^** | 99 | |
| *dagl-1(tm2908) EV* | 10.1 | -22.90 | <0.001^a^*** | 95 | |
| *dagl-1(tm2908) dgk-5(RNAi)* | 12.9 | -1.53 | 0.04 ^a^* | 96 | |
| *dagl-1(tm3026) EV* | 10.5 | -19.85 | <0.001^a^*** | 86 | |
| *dagl-1(tm3026) dgk-5(RNAi)* | 13.0 | -0.76 | 0.05 ^a^ | 68 | |
| N2 *EV* | 12.8 | - | - | 36 | |
| N2 *let-363(RNAi)* | 15.0 | 17.24 | <0.001^a^*** | 22 | |
| *dagl-1(tm2908) EV* | 10.7 | -15.84 | <0.001^a^*** | 30 | |
| *dagl-1(tm2908) let-363(RNAi)* | 13.9 | 8.12 | 0.048 ^a^* | 24 | |
| *dagl-1(tm3026) EV* | 11.5 | -10.13 | <0.001^a^*** | 30 | |
| *dagl-1(tm3026) let-363(RNAi)* | 13.9 | 8.49 | 0.036 ^a^* | 26 | |
| N2 *EV* | 12.8 | - | - | 30 | |
| N2 *let-363(RNAi)* | 15.2 | 19.06 | <0.001^a^*** | 30 | |
| *dagl-1(tm2908) EV* | 10.8 | -15.31 | <0.001^a^*** | 32 | |
| *dagl-1(tm2908) let-363(RNAi)* | 14.1 | 10.7 | 0.006 ^a^** | 30 | |
| *dagl-1(tm3026) EV* | 11.4 | -10.85 | <0.001^a^*** | 21 | |
| *dagl-1(tm3026)let-363(RNAi)* | 14.4 | 12.79 | 0.002 ^a^** | 30 | |
| N2 *EV* | 13.2 | - | - | 98 | |
| N2 *let-363(RNAi)* | 15.15 | 14.71 | <0.001^a^*** | 34 | |
| *dagl-1(tm2908) EV* | 10.8 | -18.14 | <0.001^a^*** | 42 | |
| *dagl-1(tm2908) let-363(RNAi)* | 14.1 | 6.69 | 0.085 ^a^ | 23 | |
| *dagl-1(tm3026) EV* | 11.4 | -13.63 | <0.001^a^*** | 42 | |
| *dagl-1(tm3026) let-363(RNAi)* | 14.0 | 6.23 | 0.021 ^a^* | 38 | |
| N2 *EV* | 12.8 | - | - | 36 | |
| N2 *daf-15(RNAi)* | 14.6 | 13.59 | 0.011 ^a^* | 26 | |
| *dagl-1(tm2908) EV* | 10.7 | -15.84 | <0.001^a^*** | 30 | |
| *dagl-1(tm2908) daf-15(RNAi)* | 13.8 | 7.36 | 0.045 ^a^* | 27 | |
| *dagl-1(tm3026) EV* | 11.5 | -10.13 | <0.001^a^*** | 30 | |
| *dagl-1(tm3026) daf-15(RNAi)* | 13.9 | 4.03 | 0.202 ^a^ | 30 | |
| N2 *EV* | 12.8 | - | - | 30 | |
| N2 *daf-15(RNAi)* | 14.6 | 14.41 | <0.001^a^*** | 33 | |
| *dagl-1(tm2908) EV* | 10.8 | -15.31 | <0.001^a^*** | 32 | |
| *dagl-1(tm2908) daf-15(RNAi)* | 14.0 | 9.93 | 0.016 ^a^* | 29 | |
| *dagl-1(tm3026) EV* | 11.4 | -10.85 | <0.001^a^*** | 21 | |
| *dagl-1(tm3026) daf-15(RNAi)* | 14.1 | 10.20 | 0.028 ^a^* | 29 | |
| N2 *EV* | 13.2 | - | - | 98 | |
| N2 *daf-15(RNAi)* | 14.6 | 10.52 | <0.001^a^*** | 32 | |
| *dagl-1(tm2908) EV* | 10.8 | -18.14 | <0.001^a^*** | 42 | |
| *dagl-1(tm2908) daf-15(RNAi)* | 13.7 | 4.03 | 0.169 ^a^ | 38 | |
| *dagl-1(tm3026) EV* | 11.4 | -13.63 | <0.001^a^*** | 42 | |
| *dagl-1(tm3026) daf-15(RNAi)* | 14.0 | 6.33 | 0.044 ^a^* | 25 | |
| Lifespan is carried out at 20°C. *P* values are calculated by log rank test. (**P*<0.05, ***P*<0.01, ****P*<0.001)  ^a^ Compared to N2 grown on empty vector control. | | | | |  |

| Table S10. The lifespan of N2, *dgk-5(ok2366)* and *dgk-5(gk631)* in *C. elegans*. (the three separate data for Table S6) | | | | |
| --- | --- | --- | --- | --- |
| Strain | Mean Lifespan (Days) | Difference (%) | *P*-value | n |
| N2 | 11.8 | - | - | 80 |
| *dgk-5(ok2366)* | 12.9 | 8.99 | 0.02^a^* | 88 |
| *dgk-5(gk631)* | 14.3 | 20.9 | <0.001^a^*** | 53 |
| N2 | 12.4 | - | - | 49 |
| *dgk-5(ok2366)* | 13.2 | 6.65 | 0.001^a^** | 79 |
| *dgk-5(gk631)* | 13.2 | 6.06 | 0.01 ^a^* | 77 |
| N2 | 12.7 | - | - | 87 |
| *dgk-5(ok2366)* | 13.6 | 7.73 | 0.009** | 120 |
| *dgk-5(gk631)* | 13.7 | 7.77 | <0.001^a^*** | 77 |

| Lifespan is carried out at 20°C. *P* values are calculated by log rank test. (**p*<0.05, ***p*<0.01, ****p*<0.001)  ^a^ Compared to N2 grown on *E. coli OP50*. |
| --- |

Reference:

Zhao Q, He Z, Chen N, Cho YY, Zhu F, Lu C, Ma WY, Bode AM , Dong Z (2005). 2-Arachidonoylglycerol stimulates activator protein-1-dependent transcriptional activity and enhances epidermal growth factor-induced cell transformation in JB6 P+ cells. *The Journal of biological chemistry*. **280**, 26735-26742.
