# Supplementary material for: Diacylglycerol lipase regulates lifespan and oxidative stress response by inversely modulating TOR signaling in Drosophila and C. elegans
Source: Aging Cell. 2014 May 30;13(4):755–64. doi: 10.1111/acel.12232 (PMC4116436; doi:10.1111/acel.12232)
Supplement: Supplementary file 2 [file acel0013-0755-sd2.pdf]

Table for data of *w*^{1118} male Fig. 1A

Supplementary table

age (d)	interval (d)	Nx	dx
0	n/a	273	0
4	4	273	5
7	3	268	5
11	4	263	4
14	3	259	11
17	3	248	17
20	3	231	29
24	4	202	29
27	3	173	20
31	4	153	25
34	3	128	28
38	4	100	25
41	3	75	41
45	4	34	17
48	3	17	12
52	4	5	3
55	3	2	2
59	4	0	0

Table for data of *inaE* ^{EP1101} male Fig. 1A

Supplementary table

age (d)	interval (d)	Nx	dx
0	n/a	306	0
4	4	306	2
7	3	304	0
11	4	304	1
14	3	303	2
17	3	301	0
20	3	301	0
24	4	301	1
27	3	300	2
31	4	298	5
34	3	293	2
38	4	291	2
41	3	289	4
45	4	285	6
48	3	279	15
52	4	264	18
55	3	246	28
59	4	218	27
62	3	191	72
66	4	119	28
69	3	91	59
73	4	32	5
76	3	27	14
80	4	13	11
87	7	2	2
90	3	0	0

Table for data of *w*^{1118} male Fig. 1B

Supplementary table

**age (hr)**	**interval (hr)**	**Nx**	**dx**
0	n/a	69	6
16	16	63	6
20	4	57	2
24	4	55	4
28	4	51	11
40	12	40	7
44	4	33	8
48	4	25	6
52	4	19	17
64	12	2	1
69	5	1	1
73	4	0	0

Table for data of *inaE* ^{EP1101} male Fig. 1B

Supplementary table

age (hr)	interval (hr)	Nx	dx
0	n/a	87	1
16	16	86	1
20	4	85	3
24	4	82	1
28	4	81	5
40	12	76	0
44	4	76	3
48	4	73	2
52	4	71	10
64	12	61	7
69	5	54	8
73	4	46	9
76	3	37	14
87	11	23	8
91	4	15	6
96	5	9	5
100	4	4	4
112	12	0	0

Table for data of w^{1118} male Fig. 1E

Supplementary table

age (d)	interval (d)	Nx	dx
0	n/a	300	2
3	3	298	2
7	4	296	3
10	3	293	0
14	4	293	3
17	3	290	12
21	4	278	4
24	3	274	34
28	4	240	47
35	7	193	13
38	3	180	47
42	4	133	31
45	3	102	53
49	4	49	17
52	3	32	17
57	5	15	7
60	3	8	2
63	3	6	4
66	3	2	1
70	4	1	1
77	7	0	0

Table for data of *inaE*^{KG08585} male Fig. 1E

Supplementary table

age (d)	interval (d)	Nx	dx
0	n/a	254	24
4	4	230	14
7	3	216	12
11	4	204	15
14	3	189	13
17	3	176	37
20	3	139	61
24	4	78	67
27	3	11	3
31	4	8	4
34	3	4	2
38	4	2	2
41	3	0	0

Table for data of *w* ^{1118} male Fig. 1F

Supplementary table

age (hr)	interval (hr)	Nx	dx
0	n/a	100	17
17	17	83	5
21	4	78	7
25	4	71	6
28	3	65	11
41	13	54	2
45	4	52	2
49	4	50	1
52	3	49	8
64	12	41	3
68	4	38	6
72	4	32	5
75	3	27	10
89	14	17	5
93	4	12	6
97	4	6	1
100	3	5	4
113	13	1	1
117	4	0	0

Table for data of *inaE* ^{KG08585} male Fig. 1F

Supplementary table

**age (hr)**	**interval (hr)**	**Nx**	**dx**
0	n/a	100	43
17	17	57	4
21	4	53	7
25	4	46	7
28	3	39	12
41	13	27	1
45	4	26	0
49	4	26	0
52	3	26	7
64	12	19	4
68	4	15	3
72	4	12	5
75	3	7	4
89	14	3	1
93	4	2	0
97	4	2	1
100	3	1	1
113	13	0	0

Table for data of *GMR-Gal4>UAS-DAGL* Fig. 2A,C

Supplementary table

**age (d)**	**interval (d)**	**Nx**	**dx**
0	n/a	206	0
5	5	206	0
8	3	206	0
9	1	206	0
11	2	206	1
14	3	205	2
18	4	203	0
21	3	203	1
24	3	202	3
28	4	199	5
31	3	194	17
35	4	177	18
38	3	159	25
42	4	134	23
46	4	111	25
49	3	86	21
52	3	65	16
56	4	49	26
59	3	23	9
63	4	14	5
66	3	9	3
70	4	6	5
73	3	1	0
77	4	1	0
80	3	1	1
84	4	0	0

Table for data of *UAS-DAGL/+* Fig. 2A

Supplementary table

**age (d)**	**interval (d)**	**Nx**	**dx**
0	n/a	292	0
5	5	292	0
8	3	292	0
9	1	292	2
11	2	290	1
14	3	289	4
18	4	285	3
21	3	282	7
24	3	275	15
28	4	260	18
31	3	242	34
35	4	208	71
38	3	137	34
42	4	103	18
46	4	85	23
49	3	62	18
52	3	44	13
56	4	31	9
59	3	22	10
63	4	12	3
66	3	9	5
70	4	4	2
73	3	2	0
77	4	2	2
80	3	0	0

Table for data of *GMR-Gal4/+* Fig. 2A,B,C

Supplementary table

**age (d)**	**interval (d)**	**Nx**	**dx**
0	n/a	258	0
5	5	258	0
8	3	258	0
9	1	258	2
11	2	256	6
14	3	250	6
18	4	244	16
21	3	228	25
24	3	203	26
28	4	177	32
31	3	145	27
35	4	118	39
38	3	79	46
42	4	33	14
46	4	19	7
49	3	12	5
52	3	7	4
56	4	3	0
59	3	3	2
63	4	1	0
66	3	1	1
70	4	0	0

Table for data of *GMR-Gal4>UAS-rdgA* ^{RNAi} Fig. 2B,C

Supplementary table

**age (d)**	**interval (d)**	**Nx**	**dx**
0	n/a	62	0
5	5	62	0
8	3	62	0
9	1	62	1
11	2	61	0
14	3	61	2
18	4	59	1
21	3	58	1
24	3	57	0
28	4	57	1
31	3	56	3
35	4	53	4
38	3	49	7
42	4	42	9
46	4	33	7
49	3	26	4
52	3	22	5
56	4	17	1
59	3	16	7
63	4	9	2
66	3	7	2
70	4	5	2
73	3	3	2
77	4	1	1
80	3	0	0

Table for data of *GMR-Gal4>UAS-rdgA* ^{RNAi} Fig. 2B

Supplementary table

**age (d)**	**interval (d)**	**Nx**	**dx**
0	n/a	173	0
5	5	173	0
8	3	173	2
9	1	171	0
11	2	171	1
14	3	170	0
18	4	170	1
21	3	169	8
24	3	161	6
28	4	155	12
31	3	143	16
35	4	127	27
38	3	100	18
42	4	82	11
46	4	71	12
49	3	59	20
52	3	39	11
56	4	28	17
59	3	11	2
63	4	9	3
66	3	6	2
70	4	4	4
73	3	0	0

Table for data of *GMR-Gal4>UAS-DAGL/UAS-rdgA* ^{RNAi} Fig. 2C

Supplementary table

**age (d)**	**interval (d)**	**Nx**	**dx**
0	n/a	203	0
5	5	203	0
8	3	203	1
9	1	202	1
11	2	201	2
14	3	199	0
18	4	199	4
21	3	195	5
24	3	190	3
28	4	187	6
31	3	181	11
35	4	170	19
38	3	151	25
42	4	126	14
46	4	112	16
49	3	96	28
52	3	68	14
56	4	54	20
59	3	34	17
63	4	17	10
66	3	7	5
70	4	2	1
73	3	1	1
77	4	0	0

Table for data of *da-Gal4>UAS-DAGL* Fig. 2D,F

Supplementary table

**age (d)**	**interval (d)**	**Nx**	**dx**
0	n/a	210	0
5	5	210	0
7	2	210	1
11	4	209	3
15	4	206	6
19	4	200	5
22	3	195	13
26	4	182	21
29	3	161	40
33	4	121	33
36	3	88	54
40	4	34	12
43	3	22	15
47	4	7	3
50	3	4	4
53	3	0	0

Table for data of *UAS-DAGL/+* Fig. 2D

Supplementary table

age (d)	interval (d)	Nx	dx
0	n/a	232	0
5	5	232	0
7	2	232	5
11	4	227	2
15	4	225	15
19	4	210	23
22	3	187	43
26	4	144	26
29	3	118	53
33	4	65	37
36	3	28	10
40	4	18	9
43	3	9	7
47	4	2	2
50	3	0	0

Table for data of *da-Gal4/+* Fig. 2D,E,F

Supplementary table

age (d)	interval (d)	Nx	dx
0	n/a	209	0
5	5	209	2
7	2	207	0
11	4	207	3
15	4	204	10
19	4	194	24
22	3	170	47
26	4	123	40
29	3	83	49
33	4	34	17
36	3	17	10
40	4	7	4
43	3	3	2
47	4	1	1
50	3	0	0

Table for data of *da-Gal4>UAS-S6K* ^{KQ} Fig. 2E,F

Supplementary table

**age (d)**	**interval (d)**	**Nx**	**dx**
0	n/a	50	0
5	5	50	0
7	2	50	1
11	4	49	1
15	4	48	0
19	4	48	0
22	3	48	7
26	4	41	5
29	3	36	10
33	4	26	9
36	3	17	12
40	4	5	1
43	3	4	2
47	4	2	1
50	3	1	1
53	3	0	0

Table for data of *da-Gal4>UAS-S6K*^{KQ} Fig. 2E

Supplementary table

age (d)	interval (d)	Nx	dx
0	n/a	128	0
5	5	128	0
7	2	128	2
11	4	126	8
15	4	118	16
19	4	102	29
22	3	73	26
26	4	47	15
29	3	32	14
33	4	18	11
36	3	7	6
40	4	1	0
43	3	1	1
47	4	0	0

Table for data of *da-Gal4>UAS-DAGL/UAS-S6K* ^{KQ} Fig. 2F

Supplementary table

age (d)	interval (d)	Nx	dx
0	n/a	76	0
5	5	76	0
7	2	76	0
11	4	76	1
15	4	75	1
19	4	74	3
22	3	71	6
26	4	65	17
29	3	48	20
33	4	28	13
36	3	15	12
40	4	3	1
43	3	2	2
47	4	0	0

Table for data of N2*; Ex[Pdpy-30::GFP]* Fig. 3A

Supplementary table

**age (d)**	**interval (d)**	**Nx**	**dx**
0	n/a	121	0
1	1	121	0
2	1	121	1
3	1	120	0
4	1	120	5
5	1	115	0
6	1	115	5
7	1	110	11
8	1	99	6
9	1	93	5
10	1	88	10
11	1	78	0
12	1	78	22
13	1	56	0
14	1	56	12
15	1	44	0
16	1	44	20
17	1	24	7
18	1	17	9
19	1	8	1
20	1	7	0
21	1	7	3
22	1	4	1
23	1	3	2
24	1	1	0
25	1	1	1
26	1	0	0

Table for data of N2*; Ex[Pdpy-30::dagl-1::GFP](3)* Fig. 3A

Supplementary table

**age (d)**	**interval (d)**	**Nx**	**dx**
0	n/a	104	0
1	1	104	0
2	1	104	0
3	1	104	0
4	1	104	2
5	1	102	0
6	1	102	3
7	1	99	11
8	1	88	1
9	1	87	3
10	1	84	6
11	1	78	0
12	1	78	11
13	1	67	0
14	1	67	16
15	1	51	0
16	1	51	14
17	1	37	17
18	1	20	9
19	1	11	1
20	1	10	0
21	1	10	6
22	1	4	4
23	1	0	0

Table for data of N2*; Ex[Pdpy-30::dagl-1::GFP](4)* Fig. 3A

Supplementary table

**age (d)**	**interval (d)**	**Nx**	**dx**
0	n/a	84	0
1	1	84	0
2	1	84	1
3	1	83	0
4	1	83	3
5	1	80	0
6	1	80	3
7	1	77	8
8	1	69	1
9	1	68	2
10	1	66	5
11	1	61	0
12	1	61	9
13	1	52	0
14	1	52	14
15	1	38	0
16	1	38	7
17	1	31	8
18	1	23	7
19	1	16	3
20	1	13	0
21	1	13	6
22	1	7	2
23	1	5	3
24	1	2	1
25	1	1	1
26	1	0	0

Table for data of N2 EV Fig. 3B

Supplementary table

**age (d)**	**interval (d)**	**Nx**	**dx**
0	n/a	119	0
1	1	119	0
2	1	119	0
3	1	119	0
4	1	119	0
5	1	119	1
6	1	118	2
7	1	116	3
8	1	113	12
9	1	101	15
10	1	86	14
11	1	72	14
12	1	58	11
13	1	47	13
14	1	34	11
15	1	23	6
16	1	17	3
17	1	14	6
18	1	8	4
19	1	4	3
20	1	1	1
21	1	0	0

Table for data of N2 *dagl-1* (*RNAi-1*) Fig. 3B

Supplementary table

**age (d)**	**interval (d)**	**Nx**	**dx**
0	n/a	115	0
1	1	115	0
2	1	115	0
3	1	115	0
4	1	115	3
5	1	112	4
6	1	108	3
7	1	105	12
8	1	93	14
9	1	79	14
10	1	65	17
11	1	48	14
12	1	34	10
13	1	24	12
14	1	12	3
15	1	9	4
16	1	5	2
17	1	3	2
18	1	1	1
19	1	0	0

Table for data of N2 *dagl-1* (*RNAi-2)* Fig. 3B

Supplementary table

**age (d)**	**interval (d)**	**Nx**	**dx**
0	n/a	116	0
1	1	116	0
2	1	116	0
3	1	116	0
4	1	116	3
5	1	113	3
6	1	110	3
7	1	107	7
8	1	100	12
9	1	88	21
10	1	67	16
11	1	51	15
12	1	36	13
13	1	23	6
14	1	17	7
15	1	10	5
16	1	5	1
17	1	4	2
18	1	2	2
19	1	0	0

Table for data of N2 Fig. 3C

Supplementary table

age (d)	interval (d)	Nx	dx
0	n/a	235	0
2	2	235	5
3	1	230	2
4	1	228	1
5	1	227	13
6	1	214	17
7	1	197	36
8	1	161	32
10	2	129	7
12	2	122	26
14	2	96	25
16	2	71	31
17	1	40	22
18	1	18	0
19	1	18	9
20	1	9	6
22	2	3	0
24	2	3	1
26	2	2	2
27	1	0	0

Table for data of *dagl-1(tm2908)* Fig. 3C

Supplementary table

**age (d)**	**interval (d)**	**Nx**	**dx**
0	n/a	217	1
2	2	216	2
3	1	214	13
4	1	201	4
5	1	197	30
6	1	167	26
7	1	141	29
8	1	112	26
10	2	86	14
12	2	72	36
14	2	36	19
16	2	17	10
17	1	7	0
18	1	7	1
19	1	6	4
20	1	2	2
22	2	0	0

Table for data of N2 Fig. 3D

Supplementary table

age (d)	interval (d)	Nx	dx
0	n/a	120	0
3	3	120	0
5	2	120	3
7	2	117	10
9	2	107	5
11	2	102	29
12	1	73	16
13	1	57	13
14	1	44	21
15	1	23	3
17	2	20	6
19	2	14	10
20	1	4	4
22	2	0	0

Table for data of N2*; Ex[Pdpy-30::GFP]* Fig. 3E,F

Supplementary table

**age (d)**	**interval (d)**	**Nx**	**dx**
0	n/a	108	0
1	1	108	0
2	1	108	0
3	1	108	0
4	1	108	0
5	1	108	0
6	1	108	2
7	1	106	3
8	1	103	9
9	1	94	8
10	1	86	14
11	1	72	18
12	1	54	11
13	1	43	12
14	1	31	9
15	1	22	6
16	1	16	3
17	1	13	8
18	1	5	1
19	1	4	4
20	1	0	0

Table for data of N2*; Ex[Pdpy-30::dagl-1::GFP](4)* Fig. 3E,F

Supplementary table

age (d)	interval (d)	Nx	dx
0	n/a	77	0
1	1	77	0
2	1	77	0
3	1	77	0
4	1	77	0
5	1	77	0
6	1	77	0
7	1	77	1
8	1	76	3
9	1	73	6
10	1	67	7
11	1	60	11
12	1	49	12
13	1	37	6
14	1	31	5
15	1	26	7
16	1	19	4
17	1	15	3
18	1	12	6
19	1	6	1
20	1	5	2
21	1	3	3
22	1	0	0

Table for data of *dagl-1(tm2908); Ex[Pdpy-30::GFP]* Fig. 3E

Supplementary table

age (d)	interval (d)	Nx	dx
0	n/a	93	0
1	1	93	0
2	1	93	0
3	1	93	0
4	1	93	0
5	1	93	0
6	1	93	6
7	1	87	3
8	1	84	7
9	1	77	9
10	1	68	18
11	1	50	21
12	1	29	10
13	1	19	5
14	1	14	5
15	1	9	4
16	1	5	3
17	1	2	2
18	1	0	0

Table for data of *dagl-1(tm2908); Ex[Pdpy-30::dagl-1::GFP](4)* Fig. 3E

Supplementary table

**age (d)**	**interval (d)**	**Nx**	**dx**
0	n/a	79	0
1	1	79	0
2	1	79	0
3	1	79	0
4	1	79	0
5	1	79	0
6	1	79	3
7	1	76	4
8	1	72	2
9	1	70	9
10	1	61	11
11	1	50	13
12	1	37	11
13	1	26	8
14	1	18	5
15	1	13	5
16	1	8	1
17	1	7	4
18	1	3	3
19	1	0	0

Table for data of *dagl-1(tm3026); Ex[Pdpy-30::GFP]* Fig. 3F

Supplementary table

age (d)	interval (d)	Nx	dx
0	n/a	78	0
1	1	78	0
2	1	78	0
3	1	78	0
4	1	78	0
5	1	78	0
6	1	78	4
7	1	74	4
8	1	70	5
9	1	65	10
10	1	55	15
11	1	40	17
12	1	23	7
13	1	16	3
14	1	13	4
15	1	9	4
16	1	5	2
17	1	3	3
18	1	0	0

Table for data of *dagl-1(tm3026); Ex[Pdpy-30::dagl-1::GFP](4)* Fig. 3F

Supplementary table

age (d)	interval (d)	Nx	dx
0	n/a	75	0
1	1	75	0
2	1	75	0
3	1	75	0
4	1	75	0
5	1	75	0
6	1	75	2
7	1	73	3
8	1	70	5
9	1	65	9
10	1	56	11
11	1	45	13
12	1	32	7
13	1	25	8
14	1	17	3
15	1	14	4
16	1	10	2
17	1	8	4
18	1	4	2
19	1	2	2
20	1	0	0

Table for data of N2 EV Fig. 4B

Supplementary table

**age (d)**	**interval (d)**	**Nx**	**dx**
0	n/a	377	0
1	1	377	0
2	1	377	0
3	1	377	0
4	1	377	0
5	1	377	6
6	1	371	20
7	1	351	26
8	1	325	25
9	1	300	34
10	1	266	31
11	1	235	53
12	1	182	35
13	1	147	32
14	1	115	43
15	1	72	18
16	1	54	13
17	1	41	13
18	1	28	13
19	1	15	8
20	1	7	5
21	1	2	2
22	1	0	0

Table for data of N2 *dgk-5* (*RNAi*) Fig. 4B

Supplementary table

**age (d)**	**interval (d)**	**Nx**	**dx**
0	n/a	325	0
1	1	325	0
2	1	325	0
3	1	325	0
4	1	325	0
5	1	325	0
6	1	325	6
7	1	319	9
8	1	310	12
9	1	298	19
10	1	279	25
11	1	254	27
12	1	227	59
13	1	168	39
14	1	129	30
15	1	99	39
16	1	60	16
17	1	44	13
18	1	31	12
19	1	19	12
20	1	7	5
21	1	2	2
22	1	0	0

Table for data of *dagl-1(tm2908)* EV Fig. 4B

Supplementary table

age (d)	interval (d)	Nx	dx
0	n/a	239	0
1	1	239	0
2	1	239	0
3	1	239	0
4	1	239	0
5	1	239	7
6	1	232	19
7	1	213	23
8	1	190	41
9	1	149	56
10	1	93	51
11	1	42	16
12	1	26	19
13	1	7	4
14	1	3	3
15	1	0	0

Table for data of *dagl-1(tm2908) dgk-5* (*RNAi*) Fig. 4B

Supplementary table

age (d)	interval (d)	Nx	dx
0	n/a	301	0
1	1	301	0
2	1	301	0
3	1	301	0
4	1	301	4
5	1	297	4
6	1	293	5
7	1	288	17
8	1	271	19
9	1	252	19
10	1	233	30
11	1	203	38
12	1	165	49
13	1	116	26
14	1	90	32
15	1	58	18
16	1	40	19
17	1	21	8
18	1	13	4
19	1	9	5
20	1	4	4
21	1	0	0

Table for data of *dagl-1(tm3026)* EV Fig. 4B

Supplementary table

**age (d)**	**interval (d)**	**Nx**	**dx**
0	n/a	238	0
1	1	238	0
2	1	238	0
3	1	238	0
4	1	238	0
5	1	238	16
6	1	222	25
7	1	197	24
8	1	173	35
9	1	138	33
10	1	105	27
11	1	78	34
12	1	44	17
13	1	27	11
14	1	16	10
15	1	6	6
16	1	0	0

Table for data of *dagl-1(tm3026) dgk-5(RNAi)* Fig. 4B

Supplementary table

**age (d)**	**interval (d)**	**Nx**	**dx**
0	n/a	228	0
1	1	228	0
2	1	228	0
3	1	228	0
4	1	228	0
5	1	228	1
6	1	227	11
7	1	216	8
8	1	208	21
9	1	187	16
10	1	171	20
11	1	151	20
12	1	131	42
13	1	89	26
14	1	63	19
15	1	44	14
16	1	30	16
17	1	14	6
18	1	8	5
19	1	3	2
20	1	1	1
21	1	0	0

Table for data of N2 EV Fig. 4D,E,F,G

Supplementary table

age (d)	interval (d)	Nx	dx
0	n/a	164	0
1	1	164	0
2	1	164	0
3	1	164	0
4	1	164	1
5	1	163	3
6	1	160	5
7	1	155	9
8	1	146	18
9	1	128	23
10	1	105	23
11	1	82	16
12	1	66	13
13	1	53	13
14	1	40	13
15	1	27	10
16	1	17	8
17	1	9	6
18	1	3	3
19	1	0	0

Table for data of N2 *let-363(RNAi)* Fig. 4D,E

Supplementary table

**age (d)**	**interval (d)**	**Nx**	**dx**
0	n/a	86	0
1	1	86	0
2	1	86	0
3	1	86	0
4	1	86	0
5	1	86	1
6	1	85	0
7	1	85	2
8	1	83	1
9	1	82	7
10	1	75	9
11	1	66	12
12	1	54	10
13	1	44	7
14	1	37	10
15	1	27	5
16	1	22	7
17	1	15	3
18	1	12	4
19	1	8	5
20	1	3	3
21	1	0	0

Table for data of *dagl-1(tm2908)* EV Fig. 4D,F

Supplementary table

**age (d)**	**interval (d)**	**Nx**	**dx**
0	n/a	104	0
1	1	104	0
2	1	104	0
3	1	104	0
4	1	104	0
5	1	104	3
6	1	101	8
7	1	93	11
8	1	82	22
9	1	60	23
10	1	37	25
11	1	12	3
12	1	9	7
13	1	2	1
14	1	1	1
15	1	0	0

Table for data of *dagl-1(tm2908) let-363(RNAi)* Fig. 4D

Supplementary table

**age (d)**	**interval (d)**	**Nx**	**dx**
0	n/a	77	0
1	1	77	0
2	1	77	0
3	1	77	0
4	1	77	0
5	1	77	0
6	1	77	2
7	1	75	2
8	1	73	8
9	1	65	6
10	1	59	6
11	1	53	8
12	1	45	13
13	1	32	9
14	1	23	5
15	1	18	6
16	1	12	6
17	1	6	2
18	1	4	2
19	1	2	2
20	1	0	0

Table for data of *dagl-1(tm3026)* EV Fig. 4E,G

Supplementary table

**age (d)**	**interval (d)**	**Nx**	**dx**
0	n/a	93	0
1	1	93	0
2	1	93	0
3	1	93	0
4	1	93	0
5	1	93	4
6	1	89	7
7	1	82	10
8	1	72	12
9	1	60	18
10	1	42	14
11	1	28	9
12	1	19	8
13	1	11	3
14	1	8	6
15	1	2	2
16	1	0	0

Table for data of *dagl-1(tm3026) let-363(RNAi)* Fig. 4E

Supplementary table

**age (d)**	**interval (d)**	**Nx**	**dx**
0	n/a	94	0
1	1	94	0
2	1	94	0
3	1	94	0
4	1	94	1
5	1	93	1
6	1	92	2
7	1	90	7
8	1	83	5
9	1	78	3
10	1	75	11
11	1	64	6
12	1	58	15
13	1	43	11
14	1	32	6
15	1	26	12
16	1	14	6
17	1	8	3
18	1	5	3
19	1	2	2
20	1	0	0

Table for data of N2 *daf-15(RNAi)* Fig. 4F,G

Supplementary table

**age (d)**	**interval (d)**	**Nx**	**dx**
0	n/a	91	0
1	1	91	0
2	1	91	0
3	1	91	0
4	1	91	0
5	1	91	1
6	1	90	2
7	1	88	1
8	1	87	7
9	1	80	6
10	1	74	4
11	1	70	14
12	1	56	15
13	1	41	8
14	1	33	8
15	1	25	4
16	1	21	7
17	1	14	6
18	1	8	5
19	1	3	2
20	1	1	1
21	1	0	0

Table for data of *dagl-1(tm2908) daf-15(RNAi)* Fig. 4F

Supplementary table

**age (d)**	**interval (d)**	**Nx**	**dx**
0	n/a	94	0
1	1	94	0
2	1	94	0
3	1	94	0
4	1	94	1
5	1	93	1
6	1	92	2
7	1	90	7
8	1	83	5
9	1	78	8
10	1	70	6
11	1	64	12
12	1	52	8
13	1	44	12
14	1	32	12
15	1	20	10
16	1	10	5
17	1	5	3
18	1	2	0
19	1	2	2
20	1	0	0

Table for data of *dagl-1(tm3026) daf-15(RNAi)* Fig. 4G

Supplementary table

**age (d)**	**interval (d)**	**Nx**	**dx**
0	n/a	84	0
1	1	84	0
2	1	84	0
3	1	84	0
4	1	84	0
5	1	84	1
6	1	83	3
7	1	80	3
8	1	77	6
9	1	71	5
10	1	66	9
11	1	57	7
12	1	50	10
13	1	40	7
14	1	33	16
15	1	17	8
16	1	9	6
17	1	3	1
18	1	2	2
19	1	0	0

Table for data of w^{1118} male Fig. S3A

Supplementary table

age (d)	interval (d)	Nx	dx
0	n/a	293	0
3	3	293	1
7	4	292	0
10	3	292	0
14	4	292	3
17	3	289	9
21	4	280	6
24	3	274	31
28	4	243	25
31	3	218	34
35	4	184	27
38	3	157	61
43	5	96	56
48	5	40	5
51	3	35	11
53	2	24	8
56	3	16	7
59	3	9	9
63	4	0	0

Table for data of *rdgA* ^{BL33306} male Fig. S3A

Supplementary table

**age (d)**	**interval (d)**	**Nx**	**dx**
0	n/a	279	0
3	3	279	0
7	4	279	1
10	3	278	0
14	4	278	2
17	3	276	3
21	4	273	2
24	3	271	1
28	4	270	3
31	3	267	0
35	4	267	2
38	3	265	6
43	5	259	20
48	5	239	9
51	3	230	12
53	2	218	13
56	3	205	23
59	3	182	66
63	4	116	15
66	3	101	54
70	4	47	6
73	3	41	14
77	4	27	11
80	3	16	7
84	4	9	4
87	3	5	5
91	4	0	0

Table for data of *rdgA*^{BL20320} male Fig. S3A

Supplementary table

**age (d)**	**interval (d)**	**Nx**	**dx**
0	n/a	283	0
3	3	283	1
7	4	282	2
10	3	280	2
14	4	278	0
17	3	278	2
21	4	276	1
24	3	275	3
28	4	272	5
31	3	267	10
35	4	257	7
38	3	250	21
43	5	229	25
48	5	204	19
51	3	185	10
53	2	175	17
56	3	158	21
59	3	137	48
63	4	89	11
66	3	78	33
70	4	45	7
73	3	38	21
77	4	17	10
80	3	7	7
84	4	0	0

Table for data of N2 Fig. S3D

Supplementary table

**age (d)**	**interval (d)**	**Nx**	**dx**
0	n/a	216	0
1	1	216	0
2	1	216	0
3	1	216	0
4	1	216	0
5	1	216	2
6	1	214	7
7	1	207	10
8	1	197	25
9	1	172	24
10	1	148	31
11	1	117	16
12	1	101	25
13	1	76	24
14	1	52	16
15	1	36	15
16	1	21	11
17	1	10	5
18	1	5	3
19	1	2	2
20	1	0	0

Table for data of *dgk-5(ok2366)* Fig. S3D

Supplementary table

**age (d)**	**interval (d)**	**Nx**	**dx**
0	n/a	287	0
1	1	287	0
2	1	287	0
3	1	287	0
4	1	287	0
5	1	287	3
6	1	284	6
7	1	278	13
8	1	265	21
9	1	244	25
10	1	219	33
11	1	186	31
12	1	155	29
13	1	126	25
14	1	101	20
15	1	81	24
16	1	57	22
17	1	35	12
18	1	23	9
19	1	14	4
20	1	10	4
21	1	6	4
22	1	2	2
23	1	0	0

Table for data of *dgk-5(gk631)* Fig. S3D

Supplemantary table

**age (d)**	**interval (d)**	**Nx**	**dx**
0	n/a	207	0
1	1	207	0
2	1	207	0
3	1	207	0
4	1	207	0
5	1	207	2
6	1	205	5
7	1	200	12
8	1	188	16
9	1	172	7
10	1	165	16
11	1	149	15
12	1	134	27
13	1	107	25
14	1	82	20
15	1	62	20
16	1	42	14
17	1	28	10
18	1	18	5
19	1	13	5
20	1	8	4
21	1	4	4
22	1	0	0
